# A Review on Additive Manufactured Engineering Materials for Enhanced Road Safety and Transportation Applications

**DOI:** 10.3390/polym17070877

**Published:** 2025-03-25

**Authors:** Cem Alparslan, Muhammed Fatih Yentimur, Tuba Kütük-Sert, Şenol Bayraktar

**Affiliations:** 1 Department of Mechanical Engineering, Faculty of Engineering and Architecture, Recep Tayyip Erdogan University, 53100 Rize, Türkiye; cem.alparslan@erdogan.edu.tr (C.A.); senol.bayraktar@erdogan.edu.tr (Ş.B.); 2 Department of Civil Engineering, Faculty of Engineering and Architecture, Recep Tayyip Erdogan University, 53100 Rize, Türkiye; tuba.kutuk@erdogan.edu.tr

**Keywords:** additive manufacturing, road safety barrier, polymer, metal, composite, sustainability

## Abstract

Road safety systems are critical engineering solutions designed to minimize the effects of traffic accidents and increase the safety of transportation infrastructures. Traditional road safety structures are generally manufactured using steel, concrete and polymer materials. However, manufacturing processes with these materials are high-cost, limited in terms of design flexibility and can lead to material waste. In recent years, rapidly developing additive manufacturing (AM) technologies stand out as an important alternative in the production of road safety systems. AM enables the production of complex geometries and enables the development of lightweight and high-strength structures that can absorb impact energy more effectively. This study focuses on the use of AM methods in road safety systems, examining the performance and applicability of polymer, metal and composite materials. The advantages of AM-produced road safety barriers, traffic signs, speed bumps and shock absorbing structures, depending on the material type, are evaluated. In addition, the advantages offered by AM, such as design flexibility, sustainable production processes and material efficiency, are discussed, and technical challenges and applicability limitations are also discussed. This review evaluates the current and potential applications of AM for road safety systems, providing insights into how this technology can be used more effectively in the future. The findings of the study provide significant contributions towards improving the integration of AM technologies into road safety systems from both academic and industrial perspectives. The findings of the study provide important contributions to the development of the integration of AM technologies into road safety systems from both academic and industrial perspectives. Future research can further enhance the innovative potential of AM in road safety systems, with a particular focus on sustainable material use, design optimization and energy efficiency in manufacturing processes. However, overcoming technical challenges in large-scale applications and compliance with regulatory standards are critical research areas for the widespread adoption of this technology.

## 1. Introduction

Road safety is an important interdisciplinary engineering branch that aims to prevent traffic accidents and minimize their effects. Road safety is important not only for drivers, but also for other road users such as pedestrians and cyclists. Road construction techniques, road safety systems and vehicle safety technologies are developing every day. However, traffic accidents are still one of the biggest causes of death and disability in the world. According to the World Health Organization, more than one million people lose their lives in traffic accidents every year worldwide [1]. At the same time, traffic accidents also cause great economic damage to societies. The damage caused by traffic accidents is estimated to be USD 1.8 trillion, equivalent to approximately 10–12% of the global average gross domestic product [2]. Therefore, studies on road safety are of great importance in terms of creating safer transportation systems. Road safety, which depends on factors such as drivers, other road users, vehicles, road design, road safety elements, traffic management, etc., is developing with multidisciplinary approaches every day.

Road safety elements stand out as structures that increase the safety of road users, guide drivers to stay on the road route and reduce the effects of collisions. These elements can be classified as speed bumps, warning systems, traffic signs, road markings, lighting systems and safety barriers. Road safety barriers are important in terms of helping to prevent loss of life by absorbing collision energy and preventing vehicles from leaving the road route [3]. Modern road safety barriers aim to protect pedestrians and other road users as well as ensuring the safety of drivers and passengers. Therefore, the physical, mechanical and environmental properties of the material they are made of directly affect the protection, performance and contribution of road safety barriers. Traditional safety barriers and road safety elements are mainly produced using steel, concrete and polymer materials [4]. Although these materials offer significant advantages in terms of durability, energy absorption capacity and longevity, their production processes can be costly and time-consuming. In addition, traditional production methods often lead to excessive material waste and create limitations in the design of structures with complex geometries. In this context, AM technologies, which have developed rapidly in recent years, stand out as an innovative and sustainable alternative in the production of road safety structures.

AM works with the principle of layered production, allowing the production of lightweight, high-strength, high-impact-resistant and customizable structures. This technology enables the production of complex designs with detailed internal structures that are difficult to produce with traditional methods, thus, enabling the development of road safety elements that can absorb collision energy more effectively [5,6,7]. In addition, by increasing material usage efficiency, it significantly reduces waste and speeds up production processes, making AM an attractive option in terms of sustainable production. Today, AM applications with metal, polymer and composite materials have become widespread in various road safety systems such as safety barriers, traffic signs, speed bumps, crash-absorbing structures and modular road elements. While polymer-based AM provides lightness and cost advantages, metal AM enables the production of high-strength and durable structures [8]. The use of composite materials contributes to the development of multifunctional, high energy absorption capacity and long-lasting security systems.

In this study, the current and potential applications of additive manufacturing in road safety systems are examined and the effects of different types of materials (polymers, metals and composites) on road safety structures are evaluated. In addition, the advantages offered by additive manufacturing and the technical challenges that may be encountered are discussed and perspectives on how this technology can transform road safety systems in the future are presented.

## 2. Additive Manufacturing Technologies in Road Safety Elements

**Figure 1:**
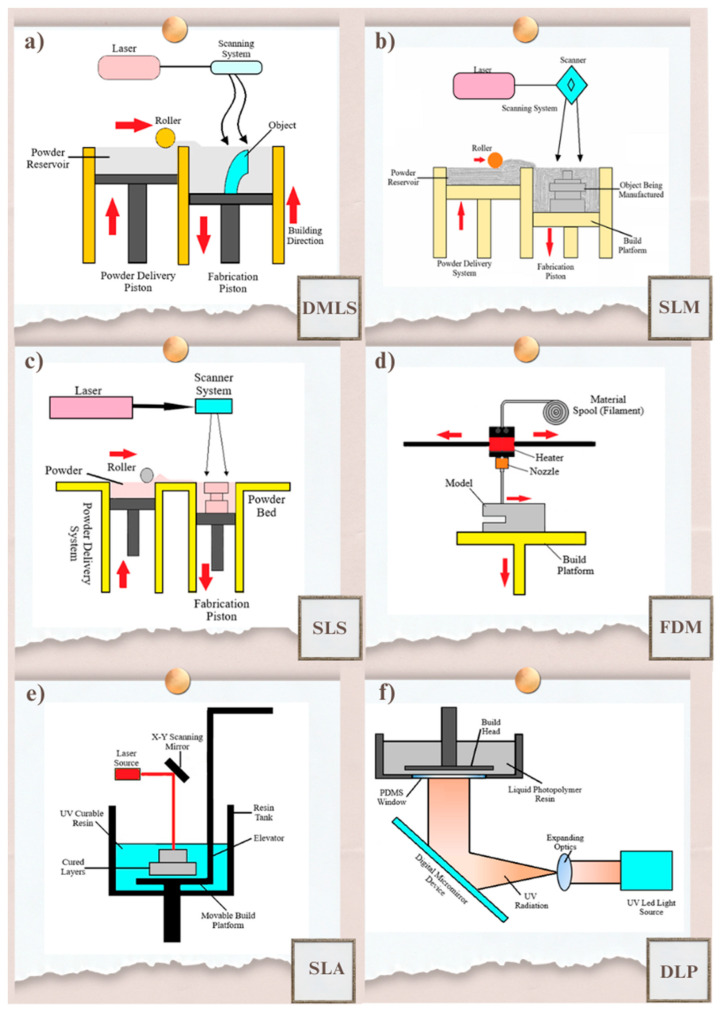
Illustration of various AM techniques and their operating principles: (**a**) DMLS, (**b**) SLM, (**c**) SLS, (**d**) FDM, (**e**) SLA and (**f**) DLP.

AM is one of the advanced manufacturing methods that combines materials in layers to produce three-dimensional structures. This technology, which was first developed for prototype production, has developed over time and has become widespread in many industries such as aviation, automotive, defense and biomedical [9,10]. AM has become an important alternative to traditional manufacturing methods thanks to its design freedom, material savings, customizable production and cost advantages. It has an important place especially in applications where parts with high strength, lightness and complex geometries need to be produced. In the aviation sector, high-performance materials such as titanium alloys and superalloys are used in the production of aircraft engine components and lightweight structural elements. The production of these parts with AM reduces the weight of the part, increases durability and contributes to fuel efficiency [11,12]. In the automotive industry, it has become possible to quickly produce special prototypes, functional parts and spare components at low cost [13]. AM is becoming increasingly common in the production of safety-focused components such as electric vehicle battery cases and bumper support elements that increase crashworthiness [14,15]. In the defense industry, AM methods are preferred to produce durable, lightweight and impact-resistant structural parts [16,17]. Resin-based methods that provide high-precision production in applications such as personalized implants, prostheses and organ models in the biomedical field come to the fore [18]. In transportation infrastructure, AM technology is considered an innovative solution in the production of road safety systems and traffic components. In the production of parts such as collision barriers, energy absorbing elements, traffic direction signs and reflector housings, lightweight and durable material compositions that increase impact resistance are used with AM. Geometric structures that are difficult to manufacture with traditional manufacturing techniques can be manufactured with AM, thus, increasing the functionality of the equipment used in road safety systems. AM processes are divided into different categories according to the types of materials used and manufacturing principles. Among the AM methods, powder bed fusion methods are based on the principle of combining a thin layer of powder with energy sources such as laser or electron beam layer by layer. These methods make it possible to produce high-performance components from materials such as metal, polymer and ceramic [19,20,21]. Direct Metal Laser Sintering (DMLS—Figure 1a) and Selective Laser Melting (SLM—Figure 1b) are processes in which metal powders are melted or sintered with the help of a controlled laser beam. DMLS allows the production of high-strength parts, especially from steel, aluminum and titanium alloys [22]. Since the SLM method provides complete melting, it is used in the production of dense and non-porous parts [23,24]. In transportation and road safety systems, aluminum and titanium-based SLM structures are widely used in the production of safety barriers that effectively absorb impact energy [25,26]. Selective Laser Sintering (SLS) produces flexible parts with high mechanical strength by sintering polymer-based powders (Figure 1c). SLS is especially preferred in the production of fasteners and modular components that require lightness and flexibility [27,28].

Fused Deposition Modeling (FDM) is based on the principle of combining thermoplastic filaments layer by layer by heating them in a controlled manner (Figure 1d). This extrusion-based method is widely preferred in prototype production because it offers a cost-effective production process. Flexible and durable structural elements can be produced with materials such as ABS, PLA and carbon fiber reinforced filaments. It is used in the transportation sector, especially in the production of modular connections, reflector covers and impact simulation components [29,30]. The integration of composite and hybrid polymers into the production process allows the production of components with high impact resistance. The FDM method plays an important role in the production of low-cost but high-strength components in road safety systems thanks to the material additives made to increase the mechanical properties of thermoplastics [31]. Another AM method, the resin-based AM method, is based on the solidification of liquid photopolymers using a light source. Stereolithography (SLA—Figure 1e) and Digital Light Processing (DLP—Figure 1f) methods offer significant advantages in the production of parts with complex geometries thanks to their high precision production capacity. SLA is preferred in the production of parts with high surface quality [32,33]. This method is used in the production of fine and detailed structures and is widely preferred in the production of optical components such as reflector lenses and signaling components. DLP works similarly to the SLA method but offers faster production and higher resolution with projection technology [34]. It is widely used in the production of traffic signal lamps and parts containing delicate details. These methods are important for the production of durable materials with optical properties. In addition, material additives to photopolymer resins increase impact resistance and provide UV resistance [35]. The use of resin-based methods in transportation safety systems such as accident simulation models and optical guidance elements enables the production of sensitive and functional components. As a result, AM is seen as an important transformation tool in today’s manufacturing world. The innovations offered by this technology have the potential to make manufacturing processes more agile, sustainable and efficient. Future research and technological advances will contribute to making AM more accessible both technically and economically, thus, enabling it to reach a wider range of industrial applications. A comparative summary of the key characteristics of the discussed AM processes is presented in Table 1. This table aims to support the reader in evaluating each method in terms of accuracy, surface quality, material compatibility, cost, production speed and practical limitations.

When AM technologies are compared with traditional manufacturing methods, it is seen that AM offers significant advantages, especially in the production of low-volume, customized parts with complex geometries [36]. In traditional methods, production is usually based on mold preparation, machining and machining processes that require high initial costs, are time-consuming and generate a significant amount of material waste during production [37]. In contrast, AM processes, thanks to their additive nature, use only the required amount of material, thereby reducing raw material waste and significantly shortening the total production time. This increases cost-effectiveness, especially in prototyping and low-volume production. From an efficiency perspective, AM methods make it possible to produce customized products quickly with less energy consumption; at the same time, they make processes suitable for automation by reducing the dependence on human intervention in the production process [5]. From a sustainability perspective, the ability to integrate recycled thermoplastics and biobased materials into production processes through AM contributes to the reduction of carbon footprint and circular economy principles [38]. With these features, AM is considered an alternative that offers lower environmental impact and greater design freedom compared to traditional methods while supporting the concept of environmentally friendly production.

## 3. Materials Used in Road Safety and Transportation Applications

**Figure 2:**
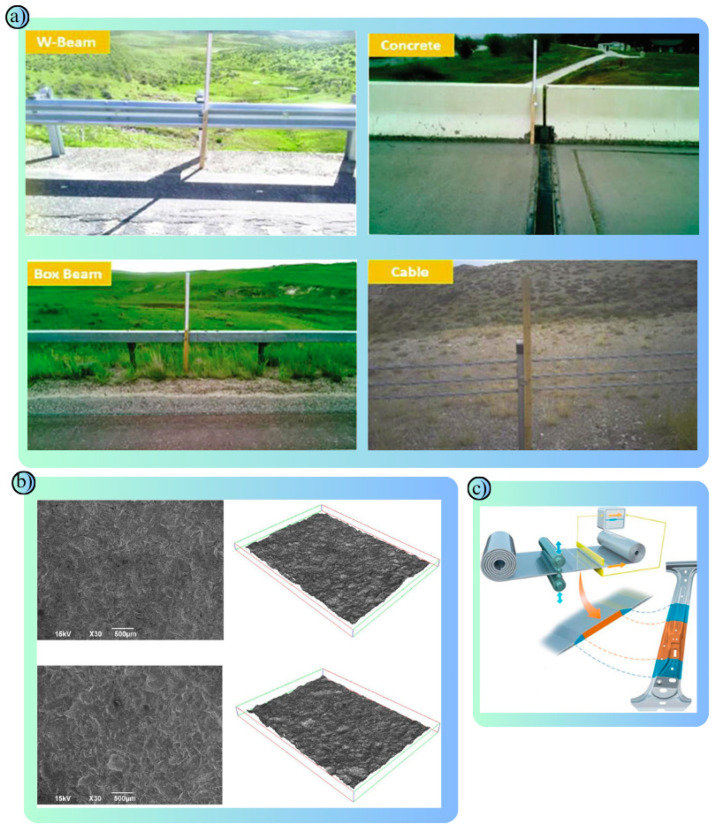
Combined figure showing various experimental and conceptual aspects of the studies together: (**a**) Different barrier systems are shown: W-Beam absorbs energy with its undulating steel structure; Concrete barrier changes vehicle direction with its rigid structure; Box Beam is structurally similar to W-Beam but has different deformation properties; Cable barrier reduces the severity of accidents by absorbing impact energy [43]. (**b**) Two different blasting methods were compared: Field blasting produces an irregular surface structure, while factory centrifugal blasting provides a more homogeneous surface. Three-dimensional models show the surface roughness and height variations [47]. (**c**) Tailor Rolled Blank (TRB) technology allows specific areas of sheet metal to be produced with different thicknesses. This method offers weight optimization while improving crash safety, especially in the automotive sector. The diagram shows the manufacturing process and its application to an automotive component [46].

As one of the most important parts of today’s modern transportation systems, road safety aims to ensure sustainability in transportation and reduce fatal/injury accidents [39]. Road safety is highly correlated not only with drivers respecting the rules, but also with vehicles, road materials, road design, traffic regulation equipment and environmental impacts. Road materials, asphalt, concrete, etc., are used to improve the strength, durability and performance of roads [40,41]. Signboards made of reflective materials and paints used in road safety lines aim to improve road visibility [42]. Road safety barriers are of particular importance in minimizing the impact of traffic accidents, reducing fatal/injury accidents and preventing vehicles from leaving the road route (Figure 2a) [43]. Road safety barriers are designed to reduce the impact of collisions, ensure the safety of drivers and other road users, and prevent vehicles from skidding off the roadway [44]. Their performance depends on the properties and type of material from which they are made. Each type of material, selected according to specific use scenarios, has different physical and mechanical properties [45]. In this study, polymers, metals and composites commonly used in road safety barriers are examined. Polymers have advantages such as corrosion resistance and their light weight. Metals are preferred due to their durability and high strength. Composites combine the advantages of these two materials to provide superior performance in terms of both energy absorption and durability. The most used metals in road safety barriers are steel and aluminum. These metals aim to reduce the impact of accidents through their energy absorption capacity and high strength (Figure 2c) [46]. However, their surfaces should be coated for corrosion resistance (Figure 2b) [47]. Polymers stand out with their flexibility, lightness and low-cost advantages and are naturally resistant to corrosion and environmental conditions [48]. Composites, on the other hand, offer a combination of a long life, high-energy absorption and light weight [49]. Composites, which are more costly than traditional materials, are increasingly preferred for their long life and low maintenance costs [50]. Used to improve road safety, these materials play an important role in protecting drivers and other road users and reducing the impact of accidents.

### 3.1. Polymers

**Figure 3:**
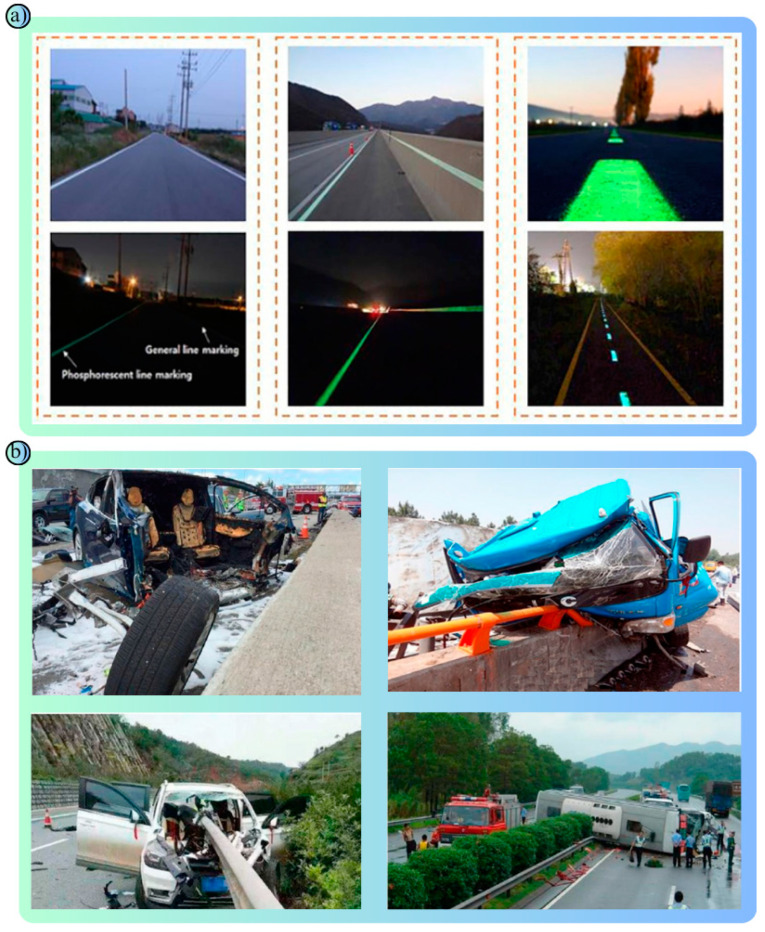
A combined figure presenting the various experimental and modeling components of the studies: (**a**) Phosphorescent road markings applied in different regions: support driving safety by improving road visibility in low light conditions. It is observed to provide better guidance at night compared to traditional lines [53,54], (**b**) Accidents with different types of barriers are shown: Concrete barriers keep vehicles on the road but can increase crash severity due to their heavy construction. W-Beam barriers pose a risk of penetration for small vehicles but can be overcome by large vehicles [51,52].

Polymers are an important material with a wide range of applications in road safety. Resistant to environmental conditions, lightweight, flexible and durable, they provide both technical and economic advantages. Polymers are preferred not only in road safety applications but also in many transportation areas such as pedestrian safety, vehicle safety, traffic management and environmental protection. For example, polymer road safety barriers (Table 1) absorb the impact of a collision, reducing damage and fatal/injury accidents (Figure 3b) [51,52]. Thermoplastic road markings are polymer-based and increase road safety by providing high visibility at road borders (Figure 3a) [53,54].

**Figure 4:**
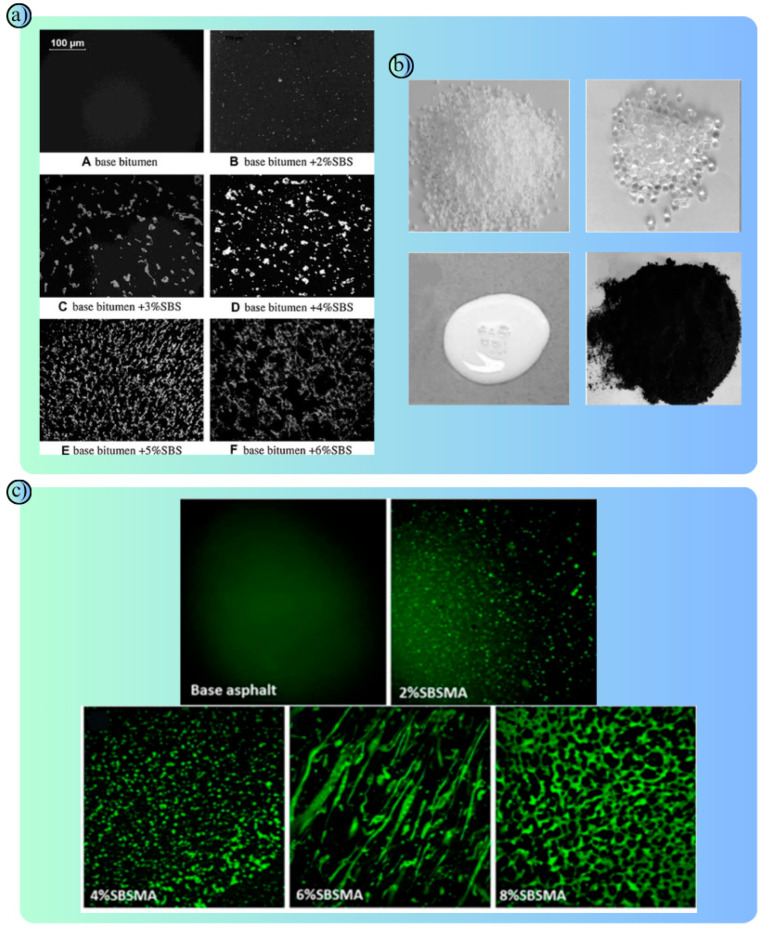
Comprehensive composite visualization representing the experimental and modeling processes of the studies in an integrated manner: (**a**) Microstructural analysis of bitumen modified with Styrene-Butadiene-Styrene (SBS): Polymer fluorescence microscope images of bitumen modified with different SBS ratios are shown. It is observed that the polymer phase distribution becomes more homogeneous and denser as the SBS content increases [55]. (**b**) Four different types of polymers used in asphalt modification are shown: SBS (Styrene-Butadiene-Styrene): Provides elasticity. EVA (Ethylene-Vinyl Acetate): Provides rigidity and high-temperature resistance. SBR (Styrene-Butadiene Rubber): Improves bonding properties. Waste Rubber: Offers the advantage of recyclability and flexibility [56,57]. (**c**) Fluorescence microscope images of asphalt modified with different SBS ratios are shown. With increasing SBS content, the polymer phase became more visible, and the elastic properties of the asphalt increased [57].

Asphalt pavements modified with polymers extend the life of the road surface, reducing rutting, cracking and deformations and improving ride quality (Figure 4a–c) [55,56,57]. In addition, energy-absorbing systems such as crash cushions and polymer coatings help prevent accidents by increasing friction on the road surface [58,59]. Signs, traffic cones and reflective materials made of polymer materials guide drivers both day and night [60]. In terms of environmental protection, polymer-based water-filled barriers and road equipment made from recycled polymers offer both environmentally friendly and economical solutions. All these advantages make polymers an important part of road safety systems. In the future, the integration of polymers with sensor technologies and the development of new climate-resistant types will offer even greater opportunities for road safety. Polymer road safety barriers are designed to prevent vehicles from leaving the road in the case of an accident (Table 2). In the case of an accident, it minimizes the damage to both drivers and passengers and other road users by absorbing the impact energy. They are durable and long-lasting. At the same time, their modular design offers portability and easy installation. This provides a quick and effective solution for temporary road arrangements. The use of polymer road safety barriers has attracted attention in recent years due to their potential advantages over traditional materials (concrete/steel). They prevent vehicles from leaving the road route and contribute to reducing the severity of accidents [61,62].

Road safety equipment made with polymers allows waste to be recycled. Recycled polymers make a major contribution to environmental protection and sustainability [63,64]. Waste polymers, which would cause great harm if thrown into the environment, are recycled and contribute to road safety. Barriers made from plastic waste directly serve waste management and sustainability goals. This process reduces both the landfill of waste polymers and the carbon footprint [65,66].

**Table 2:** Recent studies on road safety barriers produced using polymer materials.

Year	Materials	Objective	Input Parameter	Conclusion
2022	Recycled tire rubber and Polypropylene (PP) [67]	Energy and carbon analysis of recycled materials in road safety barriers	100% synthetic rubber and PP (A), 50% TRG and 50% PP (B), 50% TRG and 50% recycled PP (C). Life cycle assessment under ISO 14040/14044 [68]. Cradle-to-Gate.	Option B achieved 38% less CO_2_ and 47% less non-renewable energy consumption. Option C showed a 69% CO_2_ reduction and 86% less energy consumption.
2023	Flexible polymer barrier [69]	Develop a flexible and reusable barrier system for runaway vehicles	Simulation and full-scale crash testing with LSDYNA. Open air test field suitable for real road conditions. Santana 2000 sedan, Chinese heavy truck. 60 km/h and 120 km/h for small sedans. 60 km/h and 120 km/h for heavy trucks.	The proposed system ensured passenger safety while successfully stopping runaway vehicles. It is also cost-effective as it is reusable.
2024	Ethylene-Vinyl Acetate (EVA) foam [70]	Design optimization of roadside safety barriers	EVA foam cylinders, LS-DYNA simulations, MASH TL4 crash tests. MASH TL4 (Manual for Assessing Safety Hardware). ASTM D3575 foam compression tests. Toyota Yaris (1100 C) and Chevrolet Silverado (2270 P). 100 km/h speed and 25° impact angle.	EVA foam improved the crash performance of the barrier by reducing impact energy by 30%. Better design parameters were developed through parallel Bayesian optimization.
2023	Modular Rotating Polymer Barrier [71]	Analyzing the crash performance of the new mountable roll barrier	LS-DYNA crash simulations. China Road Safety Facilities Design Specifications. Impact speed: 100 km/h for passenger cars; 60 km/h for trucks, and impact angle: 20°.	The rotating barrier caused less damage than conventional steel barriers, increased energy absorption and improved passenger safety.
2021	Assembled Anti-Collision Barrier (Polymer Composite) [72]	Analyzing the crash impact of a new erected barrier	LS-DYNA analysis at various vehicle speeds and angles. Tested at 100 km/h and 20° impact angle. A 1.5-ton Dodge Neon passenger car and a 10-ton Ford SUT truck were used. China Road Safety Facilities Design Specifications.	The new barrier provided better energy absorption than traditional steel barriers and prevented vehicles from driving off the bridge.
2019	Water Filled Plastic Barrier (MDPE) [73]	Evaluating the crash performance of water-filled barriers	Simulations with Arbitrary Lagrange-Euler (ALE) method. EN 1317 [74] European Road Safety Standard. Impact Speed: Low speed tests: 20 km/h. High-speed tests: 40 km/h. Impact Angle: Tested at 20°, 30° and 55° impact angles.	MDPE plastic barriers were effective at low speeds (≤20 km/h) but did not provide sufficient safety at high speeds (>50 km/h).

### 3.2. Metals

**Figure 5:**
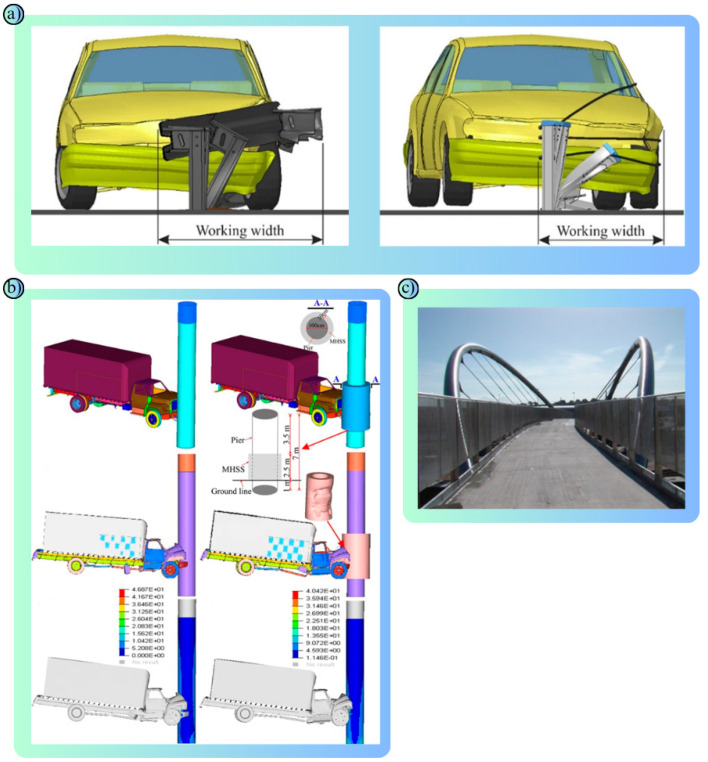
A unified visual that presents the experimental and modeling approaches of the studies in a holistic way: (**a**) The post-crash deformation behavior of different barrier designs is shown: The conventional barrier has a wider working width, while the enhanced barrier shows a more controlled deformation, helping to steer the vehicle [77]. (**b**) Simulation of bridge abutment impact in the finite element method (FEM) analysis; the impact of a truck on a bridge abutment is modeled. While the unprotected abutment is severely affected by the impact, the abutment protected with MHSS (Metal Honeycomb Safety Structure) shows lower stresses and maintains its structural integrity better [78]. (**c**) It is a modern pedestrian bridge in Holyhead, United Kingdom: Made of stainless steel, it is an important example of aesthetic and engineering design [79].

Metals have a wide range of applications in road safety due to their long life, durability and robustness. In road safety systems, metals are used in many different components, from energy-absorbing systems to barriers, road surface coatings and traffic signs. These applications of metals in transportation are designed to improve vehicle, passenger and pedestrian safety [75,76]. Steel is the most widely used metal in road safety applications. In particular, steel road safety barriers are used on highways and bridges to prevent vehicles from running off the road (Figure 5a) [77]. These barriers reduce the speed of vehicles by absorbing the impact energy and dispersing the impact of the crash, allowing passengers to survive the accident with minimal damage (Figure 5b) [78]. Thanks to their high-strength construction, steel barriers (Table 3) prevent even heavy vehicles from breaching them. It is also made resistant to corrosion by a type of coating method called galvanizing, which makes it resistant to harsh environmental conditions. Stainless steel is often used in road safety products that require long-term use and aesthetics (Figure 5c) [79]. For example, stainless steel is preferred for bridge railings and pedestrian crossings. Stainless steel materials are little affected by environmental influences and provide high performance with minimum maintenance.

Aluminum is often preferred for barrier components, directional boards and traffic signs due to its corrosion resistance and light weight [80,81]. With their visibility and reflectivity, aluminum sheets provide drivers with information that cannot be missed. At the same time, its light weight makes it easy to install and transport. In addition, aluminum is environmentally friendly as it is recyclable. Cast iron is widely used in household drainage systems, especially roadside gratings. They withstand heavy loads and allow surface water to drain safely off the road [82]. Its robust structure increases the deformation resistance of cast iron. Due to these properties, cast iron is preferred in infrastructure applications [83]. The role of metals in energy absorbing systems is very important [84]. Steel barriers and crash cushions increase the safety of vehicle occupants, pedestrians and other road users by absorbing energy on impact. Ductile dissipation of impact energy is achieved by utilizing the high flexibility and strength properties of metals [85]. In the future, the use of metals in road safety will be further enhanced by advancing smart technologies. Smart barrier systems made with metals such as aluminum and steel integrated with sensors will enable real-time data collection and analysis processes. These systems can provide instant warnings to road users by monitoring collision risks and traffic density. Furthermore, the use of innovative techniques that increase energy efficiency in the production processes of metals will contribute to reducing environmental impacts. As a result, the use of metals in road safety systems provides advantages such as longevity, high durability and energy absorption. Metals such as steel, stainless steel, cast iron and aluminum play an important role in both sustainability and safety. In the future, these materials are expected to feed into more advanced technologies to create more efficient and safer transportation infrastructures.

**Table 3:** Recent studies on road safety barriers produced using metal materials.

Year	Material	Objective	Input Parameter	Conclusion
2022	Three-wave steel beam [86]	Increase crashworthiness.	Steel profile embedded in concrete designed for crash resistance. China highway safety design standards. Eight different crash scenarios were tested. Impact Speed: 50 km/h, 60 km/h, 80 km/h and 100 km/h speed and Impact Angle: 20° impact angle.	Improved strength with new design. Maximum displacement 568.48 mm meeting Class 8 standards.
2023	Steel barrier [87]	Improving safety in freight car collisions	Three-wave design. Collision Speeds: 90 km/h and Collision Angles: 10°, 15°, 20°, 25°, 30°.	Absorbed 60% of the momentum. Reduced accident severity transferred to passengers.
2023	Q690 material [88]	Developing a passage system without a wing wall	Bridge–road barrier connection. Standard for Safety Performance Evaluation of Highway Barriers (JTG B05-01-2013) [89]. Design Guidelines for Highway Safety Facilities (JTG/T D81-2017) [90]. Collision Speeds: Small vehicle: 100.86 km/h. Medium bus: 80.75 km/h. Trailer truck: 60.58 km/h. Collision Angles: Small vehicle: 20.4°. Medium bus: 19.8° Trailer truck: 21.1°.	Successful transition reducing the risk of snagging. Absorbed 280 KJ of crash energy. SB class protection.
2023	Ti-Nb Microalloyed steel [91]	Matching microstructure properties to crash loads. Microalloyed steel analyzed by laboratory tests	Physical and mechanical properties of microalloyed steel.	Microstructure developed for crash loads. Yield strength 729 MPA, tensile strength 813 MPa.
2024	Steel flange (Figure 6a,b) [92]	Reliably measure the load-bearing capacity. Material properties were evaluated with the non-destructive Barkhausen method	Magnetic properties measured by non-destructive Barkhausen noise.	The load-bearing capacity was reliably assessed. A correlation was found between notch toughness and magnetic hardness.
2024	Crash barriers [93]	Optimizing barrier design and reducing impact in derailments of high-speed trains. Modeling with ABAQUS	Barrier used in train collision simulations. Embedded reinforcements. Code for Design of Architecture and Concrete Structures (GB50010-2010) [94]. Collision Speed: 200 km/h. Collision Angle: 5°.	Optimized design for high-speed trains. Effective in terms of dynamic damage prevention.
2022	W-beam barrier [95]	Poles in metal sleeves were evaluated.	Steel pole system supported by metal sleeves. MASH Test 3-35. Collision Speed: 64.0 mph (103 km/h). Collision Angle: 25.4°.	Comparable performance to buried poles, compliant with MASH standards.
2024	Al 6061-T6 material [96]	Increase deformation resistance and moment of inertia. Beam optimization using LS-DYNA software	Optimized aluminum alloy W-beam cross-section geometry. JTG/T F83-01-2004 [97]. Collision Speed: 100 km/h. Collision Angle: 20°.	Higher inertia and reduced deformation. Deformation reduced by 12.8%, moment of inertia increased by 28.6%.
2022	Triple beam barrier [98]	Making bridge barrier connections safe. Crash tests were conducted in accordance with MASH standards.	Midwest Barrier System. MASH Test Level 3 (TL-3). Collision Speeds: Compact: 64.6 mph (104 km/h). Pickup: 62.7 mph (101 km/h). Collision Angle: Compact: 25.2°. Pickup: 24.9°.	Safe and effective bridge barrier connection. Compliant with MASH TL-3 standards, prevents vehicle entrapment.
2024	Movable steel barrier [99]	Improving highway safety with fast opening and closing barriers.	Composite corrugated movable steel barrier mechanism. JTG B05-01-2013 [89]. Collision Speeds: Small passenger vehicle: 100 km/h. Medium passenger vehicle: 80 km/h. Large truck: 60 km/h. Collision Angle: 20°.	Fast installation for highway safety. Installation speed 12 m/min, closing speed 2 min.
2021	Cable barriers and strong post barriers [100]	Analyzing crash outcomes with cable barriers and pole barriers. Data analysis was performed with mixed logit models.	Analysis of cable barriers and strong post barriers.	Cable barriers have the lowest share of fatal/injury accidents with 24%.

### 3.3. Composites

**Figure 7:**
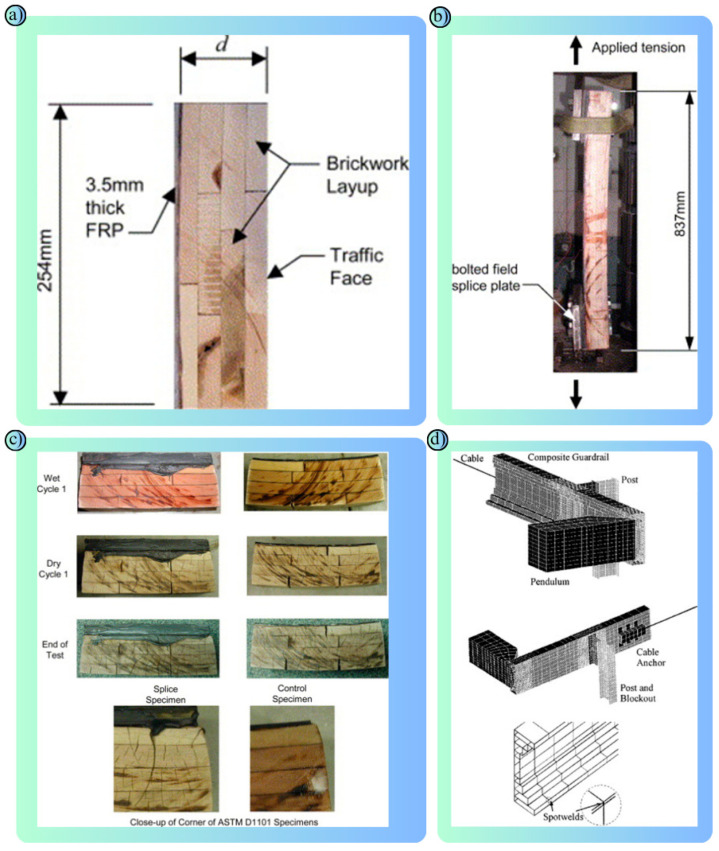
Comprehensive schema that integrally visualizes the experimental analyses and modeling components of the studies: (**a**) FRP-Glulam Barrier Section: 3.5 mm FRP cladding and a cross-section of a barrier with laminated timber in a brick pattern are shown [101]. (**b**) Tensile Test Specimen: The 837 mm long specimen was subjected to a tensile test with a bolted connection plate. Strain gauges were used to monitor the maximum stresses on the FRP [101]. (**c**) ASTM D1101 Test Results: Specimens exposed to moisture and drying cycles showed no delamination in the steel-reinforced specimens, while small cracks were detected in the control specimens [101]. (**d**) LS-DYNA Composite Barrier Model: The crash behavior of the barrier was modeled using the finite element method (FEM). Spotwelds were used to connect the composite parts [102].

Composite materials have an important place in the field of road safety with their flexibility, lightness, durability and resistance to corrosion (Figure 7a–c) [101]. A composite is a material formed by combining two or more materials. High performance is achieved by combining the best properties of each material. In road safety, composites are particularly used in road safety barriers, energy-absorbing systems, crash cushions, road markings and structural reinforcement applications (Figure 7d) [102].

Composites are used to reinforce structural elements, increase the durability of road markings, reduce the impact of collisions and prevent vehicles from running off the road. They are used in road safety barriers and crash cushions due to their lightweight and high energy absorption properties. The use of composites in road safety barriers (Table 4) minimizes deformation and allows the barriers to be reused after a crash. Composites such as composite-steel barriers, glass fiber-reinforced plastic (GFRP) or carbon fiber-reinforced plastic (CFRP) absorb crash energy in systems such as barriers and crash cushions and make the system reusable.

**Figure 8:**
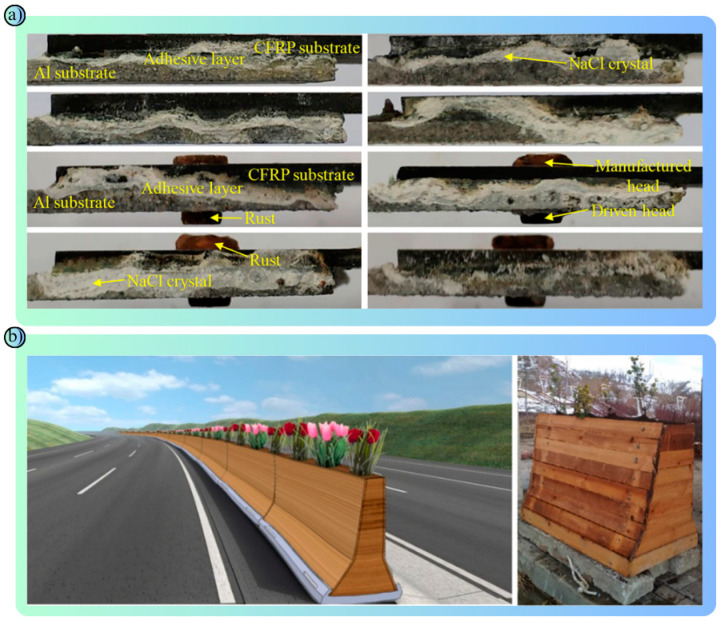
Integrated display that presents the experimental findings and modeling approaches of the studies in a holistic perspective: (**a**) Corrosion Damage to CFRP/Al Connections: CFRP provided protection against corrosion. NaCl crystals and rust were concentrated in the adhesive layer and at the rivet heads. While corrosion was more limited in areas covered with CFRP, significant galvanic corrosion occurred between the steel rivet and the Al substrate. This indicates that CFRP improves corrosion resistance but requires additional protection at the metal joint areas [111]. (**b**) A design visualization and prototype of a hybrid traffic barrier consisting of a concrete base, steel profiles, wood panels and vegetation is presented: This design aims to enhance environmental sustainability and aesthetic harmony while ensuring traffic safety [118].

Due to their light weight and corrosion resistance, composites are also used in road marking signs. Composite plates, which are a combination of polymer and aluminum, have a longer life compared to traditional materials. In addition, reflective coatings can be applied to composite-based surfaces to provide drivers with night vision comfort. GFRP, a composite of glass fiber and polymer, is a widely used composite in road safety systems. It is both economical, durable and lightweight. Used in road safety barriers, traffic signs and drainage coatings [103,104]. Offering higher flexibility and strength than glass fiber, CFRP is used in energy-absorbing barriers, crash cushions and bridge reinforcements thanks to its high energy absorption capacity. CFRP is also highly resistant to chemical effects and corrosion (Figure 8a) [105,106,107,108,109,110,111]. Kevlar-reinforced composites are preferred for pedestrian protection systems due to their high impact resistance. Kevlar-reinforced composites, which have a light and flexible structure, are also suitable for high-performance road safety systems [112]. Thermoplastic matrix composites produced by reinforcing polypropylene and polyethylene matrices with fibers are used in temporary barriers and traffic cones used in road management [113,114]. Aluminum composite panels are a combination of aluminum and polymers. Thanks to their lightweight and durable structure, they are used in directional boards, road signs and road safety barriers. They are also long-lasting and aesthetic [115,116,117].

**Table 4:** Recent studies on road safety barriers produced using composite materials.

Year	Material	Objective	Input Parameter	Conclusion
2024	Glass fiber/LDPE composite [119]	Production and characterization for W-beam barrier	E-glass fiber-reinforced LDPE ASTM D3039 [120] (for tensile tests), ASTM D6641 [121] (for compression tests)	Glass fiber-reinforced barriers have better energy absorption capacity compared to the steel alternative.
2023	Natural stem (Stipa tenacissima) composite [122]	Increase impact energy absorption	Epoxy and Alpha 10% material BS EN 1317-2:2010 [123] (impact tests for road safety barriers), ASTM E23-18 [124] (Charpy impact test) Collision Speed: 110 km/h	The composite barrier absorbed 67.7% more impact energy than steel.
2021	Basalt fiber reinforced polymer [125]	Improving crash performance	BFRP pipes, LS-DYNA simulation JTG B05-01-2013 [89] Collision Speeds: 100 km/h for passenger cars, 60 km/h for trucks	It absorbed more energy than steel barriers, keeping vehicle acceleration under 200%.
2022	Composite blocks [126]	W-beam barrier height increase	28-inch W-beam barrier system MASH Test 3-11 Collision Speed: 62 mph (100 km/h), Collision Angle: 25°	MASH Test 3-11 standard was found compliant, passenger safety was increased.
2023	Composite-steel double-layer barrier [127]	Increasing the crashworthiness of bridge barriers	Double-layer rigid-flexible barrier JTG/T D81-2017 [90] Collision Speeds: 100 km/h, 80 km/h, 60 km/h, Collision Angle: 15°, 20°, 30°	The double-layer barrier provided energy absorption equivalent to 59.1% of the concrete barrier.
2023	Carbon/Epoxy and Fiber Metal Laminated [128]	Compare the impact resistance of different barriers	Steel, composite, FML plates	The FML-B barrier was found to have the highest energy absorption capacity.
2023	Renewable hybrid barrier (RHB) [129]	Impact resistance of innovative hybrid barrier systems	Wood, steel and sand components EN 1317 [74] (TB11 Test Standard) Collision Speeds: 100 km/h. Collision Angle: 20°	The hybrid barrier was found to be as safe as steel and as comfortable as concrete.
2022	Hybrid barrier with waste materials (Figure 8b) [118]	To investigate the usability of waste materials in barriers	Wood, rubber, slag EN 1317 [74]	An environmentally friendly barrier compliant with EN 1317 tests was proposed.
2024	Recycled foam concrete [130]	Increasing energy absorption in road barriers	Thin-walled steel and foam concrete JTG B05-01-2013 [89] Collision Speed: 29.55 km/h. Collision Angle: 20°	Reduced impact force by 54.46%, reduced cost by 5.5%.
2021	Steel-concrete composite beam [131]	Analyzing the effect of parapets	Simple beam composite bridge model	A 26.92% lower load distribution was detected with the parapet effect.

**Figure 9:**
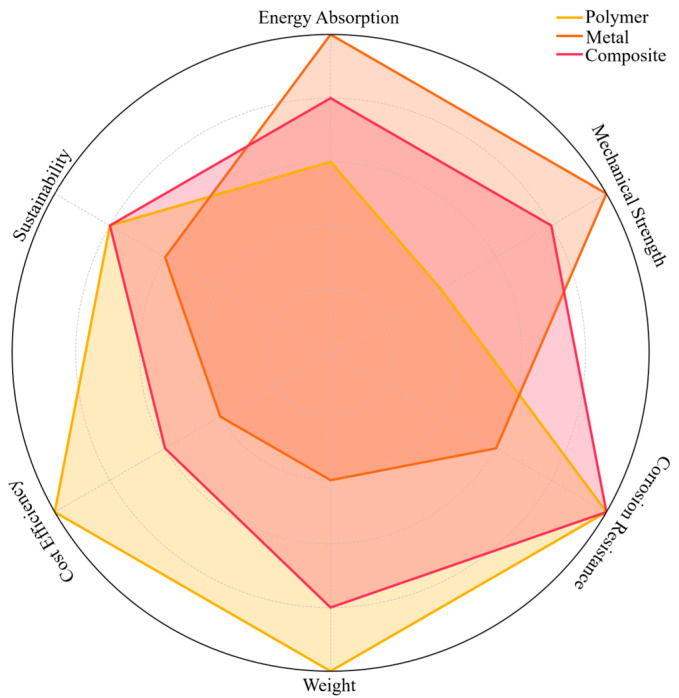
Comparative evaluation of material types in road safety systems.

The polymer, metal and composite materials examined in this study offer various advantages in terms of different performance criteria in road safety systems. Polymer materials stand out especially in temporary safety applications due to their light weight, low cost, corrosion resistance and easy portability. However, they have some limitations in terms of impact resistance and longevity. Metal materials are widely used in permanent security barriers due to their high mechanical strength, energy absorption capacity and long-term durability. However, they have disadvantages such as weight, cost and corrosion risk. Composite materials, on the other hand, combine the advantages of polymer and metal-based structures and offer superior properties such as high energy absorption capacity, light weight, corrosion resistance and long life (Figure 9). However, their widespread use is still limited due to high production costs and complex manufacturing processes. As a result, each type of material is suitable for different usage scenarios, and the optimum choice of material for road safety systems should be made by considering the application location, environmental conditions, economic factors and performance expectations.

## 4. AMed Materials and Its Road Safety and Transportation Structures

AM technologies have become widely used in various industrial applications in recent years due to their advantages such as design flexibility, material savings and easy production of complex geometries [132,133,134,135]. These advantages have increased the use of AM methods in areas such as defense, aviation, automotive and medicine, and have recently begun to be seen as a potential solution in infrastructure projects. In particular, design freedom allows for the production of structures optimized to meet performance requirements by allowing structural details that cannot be produced with traditional methods to be obtained [136,137]. In this context, AM stands out in the production of materials that must have critical properties such as strength, flexibility, energy absorption and lightness, such as road barriers, crash cushions, safety parapets, vehicle bumpers and aircraft equipment [138,139]. Absorption of kinetic energy and controlled deformation at the time of collision is one of the basic functions of barrier systems. Therefore, the applicability of different types of materials used in AM in road barrier systems has become an important area of research. Road barriers are structural elements designed to ensure the safe direction of traffic and to reduce the effects of collisions [140]. These structures must offer high mechanical strength and energy absorption capacity, as well as being economically viable. In barrier designs for different road types, especially urban and highway, modularity and easy installation features are gaining importance along with cost-effectiveness. Since barrier systems produced with traditional production methods are generally heavy and require maintenance, it is of great importance to ensure the balance between lightness and durability [141]. It is a common problem that barriers manufactured with traditional materials such as steel and concrete to achieve this balance deform over time or lose performance due to environmental conditions [101]. AM stands out as an innovative production method that can meet these requirements. Thanks to the layered production approach offered by AM, material density and structural durability can be optimized in different areas. This enables the use of stronger structural elements in areas requiring energy absorption in barrier systems, and thinner and lighter structures in areas requiring lightness. In addition, geometric structures can be tested with simulation-supported optimization methods in design processes and performance improvements can be made.

As a result, road safety structures that can be produced with AM technologies offer an innovative solution in future infrastructure projects not only with their mechanical strength and lightness, but also with their advantages such as customizability, fast production processes and ease of maintenance [142]. Advances in this area will continue to contribute to the development of safer and more durable barrier systems.

### 4.1. Polymer-Based Materials

**Figure 10:**
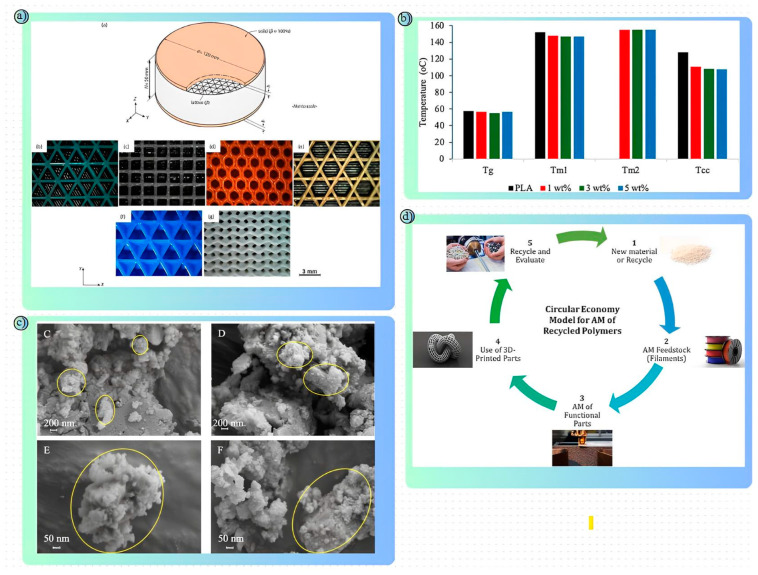
Merged plot showing various experimental and conceptual aspects of the studies: (**a**) Lattice-based test specimen geometry and optical microscopy images of various 3D printed lattice structures (Triangle, Diamond, Honeycomb, Star, Cubic, and Gyroid) highlighting structural differences [148]. (**b**) Differential Scanning Calorimetry (DSC) results showing thermal transitions (Tg, Tm1, Tm2, and Tcc) for PLA and PLA/ESP composites at various weight percentages [152]. (**c**) SEM image of PLA/ESP composite showing nanostructural details of embedded particles [152], and (**d**) proposed circular economy model for recycled polymers in value-added manufacturing, showing the life cycle from material preparation to part reuse and valorization [153].

Polymer-based materials are a widely preferred material group in AM technologies and offer many advantages in road safety structures. Thanks to their lightness, corrosion resistance and high energy absorption capacity, polymers can absorb kinetic energy in the event of a collision, distribute deformation in a controlled manner and increase safety performance in structural elements such as barriers and crash cushions. In addition, the modular structure of polymers contributes to the development of easily transportable and mountable safety systems by providing cost-effective solutions in production and installation processes. In addition, improving the resistance levels of polymer materials to temperature changes and external environmental conditions is important for the longevity of safety structures [143,144]. In particular, high temperature differences can negatively affect the mechanical properties of some polymers. For this reason, the use of polymer composites with AM technologies has become increasingly important. Polymer composites offer higher strength, heat resistance and impact absorption capacity compared to traditional polymers thanks to their reinforcement materials [145,146]. The design flexibility offered by AM allows for the optimization of material density and structural strength locally. In this way, areas requiring energy absorption in road barrier systems are reinforced with thicker and more durable structures, while areas requiring lightness can be produced with thinner layers [147]. Thus, safety structures offer an innovative solution in future infrastructure projects not only in terms of mechanical strength and lightness, but also with advantages such as customizability, rapid production and ease of maintenance. In this context, literature studies that include the use of polymer materials with AM methods provide important findings that reveal the potential of materials and production methods that can be used in the production of equipment in road safety structures. In this study conducted by Alqahtani et al., the effects of cage topology on the thermal and mechanical performance of polymer cage structures produced by AM were investigated. In the study, different cage designs aimed at increasing energy efficiency and load-carrying capacity for structural applications were examined. Layered manufacturing was carried out using polylactic acid (PLA) filament. Cage structures were produced with the FDM method and designs were made with CAD software. Triangular, diamond, honeycomb, star, cubic and gyroid-type cage structures were examined (Figure 10a). Thermal Conductivity (K-value) was measured with a heat flow meter. Thermal Transmittance (U-value) was determined with a hot-box calorimeter. Mechanical properties were evaluated with a compression test. As a result of the study, the U-value and K-value changed according to cage density. The K-value increased by 53% in the transition from 20% density to 80% density. Triangular and diamond lattice structures exhibited optimum thermal and mechanical performance at 40% density. Triangular and diamond structures showed compressive strength above 27 Mpa. Gyroid and cubic structures exhibited high performance in energy absorption capacity, but lower values were recorded in initial strength. The study provides basic information for the design of lattice structures with high mechanical strength and low thermal conductivity that can be used in many applications. Triangular and diamond topologies in particular stand out in terms of ease of production and energy efficiency [148]. Jikke de Winter et al. investigated the damping behavior of microbeams produced using the two-photon polymerization (2PP) method. This study aimed to evaluate the potential of microresonators for use in biosensor and microsystem applications by improving their quality. IP-S resin was used in the Nanoscribe Professional GT device. This resin was preferred due to its high elastic modulus. Thick and thin type beams, cantilever and double-ended bridges were produced. The resonance frequencies and quality factors of the structures were measured using laser Doppler vibrometry (LDV). The tests were carried out at room temperature and in a high vacuum environment (≤5 × 10⁻^5^ mbar). As a result of the study, the quality factor of the 200 µm-long bridges increased from 522 to 1819 after thermal treatment. The increase in the resonance frequency in the thin bridges was attributed to the stress induction caused by thermal treatment. Structural losses were found to be the main source of damping. Thermal elastic damping and anchorage losses did not show a dominant effect. This study successfully used the thermal strain engineering approach to optimize the quality factors of 3D-printed polymer microresonators. With a quality factor of 1819, these microresonators are promising for high-precision measurements in mass sensors and biomolecular detection devices. However, more extensive investigations with different material types and manufacturing parameters are recommended [149]. Md Aminul Islam et al. have examined the role of AM technology in polymer production over the last thirty years, emphasizing the advantages of AM such as design flexibility, reduction of material waste and rapid prototyping. In the study, a comparative analysis of AM techniques such as FDM, SLA, PolyJet, SLS and LOM was made; SEM, TGA, DSC and DMA tests were applied using polymers such as PLA, ABS and polyamide and composite materials. It was stated that AM is used in areas such as biomedical implants, aerospace parts and industrial components. It provides up to 40% material savings and shortens the production time of structures with complex geometries by 50%. In thermal analyses, the glass transition temperatures and mechanical strength values of thermoplastic materials such as PLA were compared for different printing techniques. Studies conducted especially on multi-material structures and hybrid composites have shown the success of AM in producing customizable functional parts. As a result, AM is predicted to revolutionize sustainability and production efficiency in the aerospace, automotive and medical sectors and will further evolve with AI-assisted design [150]. Amin Safi Jahanshahi et al. investigated the simultaneous impregnation extrusion-based AM process to increase the compressive strength of PLA composites reinforced with continuous glass fibers (CGF). In the study, continuous fibers were fed into the molten polymer through a side nozzle and placed in layers on the substrate. In the compression tests, the alignment of the glass fibers parallel and perpendicular to the load direction was evaluated. The test results showed that parallel alignment of the fibers in the PLA matrix caused delamination and provided a strength of 40.7 Mpa, while perpendicular alignment increased the strength up to 93 Mpa. The perpendicular orientation of the fibers increased the mechanical strength by approximately 10.5% by creating transverse stresses against the applied load. SEM analysis showed that the crack propagation in the PLA matrix was concentrated especially in the corner regions and separation occurred at the fiber-matrix interface under load. These results reveal that structures with high energy absorption capacity can be used to increase crashworthiness in the automotive industry [151].

In a study conducted to improve the thermomechanical properties of polylactic acid (PLA) composites reinforced with eggshell microparticles (ESP), David K. Orisekeh et al. produced and characterized PLA filaments with ESP additives at different weight ratios (0%, 1%, 3% and 5%) via extrusion. XRD and FTIR analyses showed that the eggshell powder contained high-purity calcium carbonate (CaCO_3_). In TGA and DSC tests, it was observed that the melting temperature of the PLA sample with 5% ESP additive decreased from 152 °C to 147 °C, but the crystallization rate increased (Figure 10b,c). According to the dynamic mechanical analysis (DMA) results, the samples with 3% ESP additive reached the highest storage modulus with a 5.2% increase at room temperature. The compressive strength of the composites was measured as 66.5 MPa in the sample with 5% ESP additive. However, it was observed that the eggshell additive provided limited improvement in mechanical strength in tensile and flexural tests. As a result, it was stated that ESP additive partially improved the thermomechanical properties of PLA and is promising for biomedical applications. The study suggested finer particles and improved extrusion processes to increase the homogeneity of the filler distribution [152]. Alianna Maguire et al. investigated the mechanical properties and application areas of polymer-based structures used in extrusion-based AM. The study highlighted the flexibility and low-cost advantages offered by extrusion-based techniques such as FDM and direct ink writing (DIW) for the production of high-performance structures in the automotive, aerospace, medical and electronics sectors. In particular, multi-material structures consisting of thermoplastic and thermoset materials were produced with the DIW technique and the effects of different geometries on mechanical strength were examined. Different architectures increased the load-carrying capacity of the structures and improved their energy absorption properties. Composite structures to which reinforcement materials such as carbon fiber were added exhibited twice the mechanical strength compared to standard polymers. The study stated that biomimetic designs allow innovative structures to be created in AM and that these processes open new horizons in areas such as biomedical implants and energy-absorbing structures. In the future, it is suggested that production processes will be improved with artificial intelligence-supported design and better optimization of rheological properties [154]. S.C. Daminabo et al. investigated FDM-based AM processes for polymer material systems and evaluated the effects of different parameters on mechanical, thermal and surface properties. Different filament types such as PLA, ABS, PETG and TPU were used in the study; the effects of factors such as printing temperature, speed, layer height and filling ratio on performance were analyzed. According to the findings, PLA showed high compressive and tensile strength but exhibited brittleness, while ABS offered superior performance in impact resistance thanks to its flexible structure, but its layer bonding strength was found to be low. TPU stands out as a material with high energy absorption capacity and elasticity, while PETG’s high glass transition temperature increased its thermal stability. Reducing the layer height increased surface smoothness, but extended the production time. When the filling ratio was changed from 50% to 100%, the mechanical strength increased by 35% and the production time was extended by 70%. It was emphasized that porous structures are promising for cellular proliferation in biomedical applications, but biocompatible filaments need to be developed further. The research revealed that FDM-based manufacturing processes can be further improved with the use of carbon fiber-reinforced filaments and multifunctional material systems [155]. Ans Al Rashid and Muammer Koç conducted a study on the use of recycled polymeric waste in AM and evaluated the needs, challenges and opportunities of this approach within the framework of circular economy and sustainability (Figure 10d). The study stated that 8.3 billion tons of plastic have been produced worldwide since 1950 and a large part of this cannot be recycled. It was emphasized that mechanical, chemical and thermal recycling methods are used in the recycling of polymers; however, factors such as separation difficulties, additives, coatings and contamination make recycling difficult. While mechanical recycling stands out as a low-cost and environmentally friendly method, problems such as quality loss and microparticle formation have been identified. Chemical recycling, on the other hand, provides high-purity polymer production, but it is an energy-intensive and expensive process. The study showed that the use of recycled polymers in AM is possible; it was stated that polymers such as PET, ABS, PLA and PP can be evaluated in rapid prototyping and functional part production. The researchers emphasized that local recycling and AM projects initiated at laboratory scale would be effective in reducing the amount of waste and drew attention to the importance of cooperation between states, industry and research institutions to achieve sustainability goals in the future [153].

### 4.2. Metal Materials

**Figure 11:**
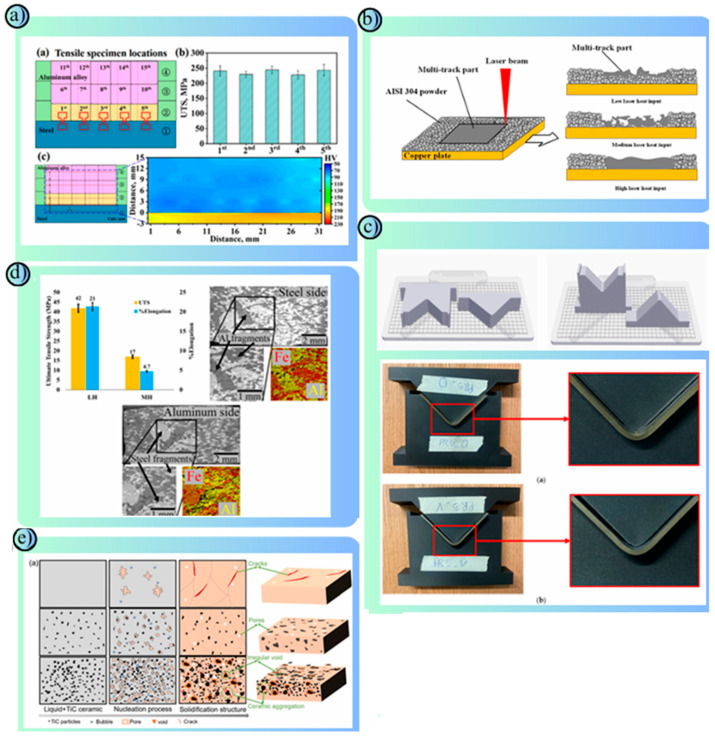
Composite figure showing multiple experimental and modeling aspects of the studies: (**a**) Tensile specimen locations and results: Tensile strength distribution along the aluminum–steel composite with hardness mapping for cross-sectional analysis [165]. (**b**) Schematic of multi-piece SLM process: Variations in laser energy heat input affecting the formation of multi-piece parts [168]. (**c**) Deformation analysis of 3D printed tools: Comparison of horizontal and vertical printed tools after bending process [169]. (**d**) Tensile testing and fracture analysis: UTS and elongation of LH and MH specimens, together with SEM images and EDS mapping showing the material distribution on the fracture surfaces [171], and (**e**) effect of TiC ceramic content: Schematic showing the mechanism of cracks, pores, and void formation during solidification in TiC ceramic composites [172].

Metal materials used in road safety structures in AM have an important place thanks to their superior mechanical strength, energy absorption capacity and long-lasting usage features [156]. While steel and steel alloys are frequently preferred in barrier systems due to their high strength and energy absorption capacity, types such as stainless steel provide advantages in terms of corrosion resistance [157,158]. Aluminum alloys stand out particularly in crash buffer systems and modular barriers with their properties such as lightness and high corrosion resistance [159]. Titanium alloys, which stand out with their high strength-to-weight ratio and chemical resistance, are used in the production of critical load-carrying fasteners [160]. Nickel-based superalloys offer a suitable option for security structures exposed to heavy traffic loads thanks to their wear and high-temperature resistance [161]. AM methods allow for topology optimization of metal materials and the production of complex geometries, reducing the weight of structures and increasing material efficiency. Metal structures produced with methods such as laser metal deposition (LMD) and selective laser melting (SLM) in particular increase the effectiveness of road safety systems by meeting high-impact resistance and long-lasting performance requirements [162,163]. Thus, metal safety structures produced with AM technologies contribute to modern traffic safety standards by providing sustainable and cost-effective solutions. In this context, metal safety structures produced with AM technologies contribute to modern traffic safety standards by providing sustainable and cost-effective solutions. Comprehensive studies are conducted in the literature on metal materials used in AM. These studies reveal the performance properties of materials that can be used in road safety structures and show that research in the field continues uninterruptedly. Peng et al. investigated the effects of C, Mn and Cu alloy elements on the microstructure and mechanical properties of high manganese steels in the wire arc additive manufacturing (WAAM) method. In the study, samples with fully austenitic microstructure were produced using powder filler wire and the effect of the contribution of the compositions on the material strength was analyzed. The experiments were grouped according to different C (0.49%, 0.79%, 1.10%), Mn (17.95%, 20.98%, 23.98%) and Cu (0.3%, 0.98%, 2.00%) ratios. The results showed that with the increase in C content, the tensile strength increased from 731 MPa to 897 MPa and the elongation increased from 21.8% to 30.2%. As the Mn content increased, the tensile strength decreased but the elongation increased. The best impact toughness value was recorded as 64.5 J at 20.98% Mn content. The increase in Cu content increased the yield strength and elongation; the highest yield strength was obtained as 495 MPa at 0.98% Cu. The study revealed that the best mechanical properties were obtained with the optimal composition of 1.10% C, 21% Mn and 0.3% Cu and that the structure was suitable for production via WAAM with low solidification crack sensitivity. It was stated that high manganese austenitic steels have promising applications in areas such as aviation, petrochemicals and rail transportation [164]. Zhang et al. aimed to increase the bond strength by producing large-scale steel-aluminum (Fe-Al) components via modified friction stir additive manufacturing (M-FSAM). Mg-O and Al-Fe-Si rich amorphous layers (~20 nm and ~120 nm) were developed to prevent the formation of brittle intermetallic compounds at the Al-Fe interface. It was observed that the thermal stability of the amorphous layer was maintained after heat treatment (20 min at 500 °C and 7 h at 170 °C). High homogeneity was achieved both along the interface and material layers by using a 2 mm overlap length with the M-FSAM method. The tensile strength increased from 160 MPa to 250 MPa and the fractures occurred on the aluminum alloy side. In addition, an increase in hardness (~80 HV) was observed after heat cycling (Figure 11a). As a result, the presence of the amorphous interlayer and the controlled application of heat treatment provided high mechanical strength and stability. This method has the potential to be used in large-scale engineering applications such as train and ship production [165]. Wessels et al. developed the Stabilized Optimal Transportation Meshfree (OTM) method to model the fusion process of metal particles using Selective Laser Melting (SLM). The main purpose of the study was to investigate the phase changes that occur during melting and cooling and the coalescence processes of metal particles. Physical phenomena such as surface tension, phase change stress, thermal expansion and recoil pressure affecting particle coalescence in the SLM process were modeled. In the simulations, the heating and melting of two metal particles with a laser were analyzed. Thanks to the OTM method, calculations of complex boundary surfaces were performed more accurately compared to traditional mesh-based methods. It was observed that surface tension accelerates the fusion of particles and increases the quality of the coalescence. The effect of laser power and cooling conditions on the coalescence bond was evaluated with a parametric study. The results showed that laser power and cooling parameters are decisive in the fusion quality and coalescence does not occur without surface tension. This method is considered a promising computational tool for determining optimum process parameters, especially in SLM processes [166]. Wawryniuk et al. examined the wide-ranging applications of 3D printing technology in the aerospace, automotive, and space industries. The study evaluated multiple AM technologies (e.g., FDM, SLA, DLP, SLS, SLM) and discussed the advantages of these methods in various sectors such as prototyping, functional parts production, and component repair. Three-dimensional printing offers unparalleled design freedom, especially in the production of lightweight structures with complex geometries. The study emphasized that metal-based 3D printing increases fuel efficiency and saves energy. In addition, high-impact structures were obtained in the aerospace industry using lattice structures and TPMS (Three-Way Periodic Minimal Surface) systems. In the automotive sector, the advantages of rapid prototyping of individually designed parts and low-volume production processes were emphasized. For example, components produced via 3D printing in a hypercar reduced the vehicle weight by 20%. The paper also focuses on the integration of AM technology into sustainable manufacturing practices and the environmental benefits arising from its combination with Industry 4.0. However, it is noted that challenges such as material properties, post-production processes and production scalability need to be overcome. The study highlights the innovative potential of 3D printing technology in the transportation sector, while suggesting that further research is needed to address future regulatory hurdles and material improvements [167]. Hwang et al. investigated the production of metal mesh structures using AISI 304 stainless steel powder via selective laser melting (SLM). In the study, the production process of thin-layer mesh structures was optimized using a copper base plate with high thermal conductivity. During the experiments, the effects of parameters such as laser scanning speed, powder layer thickness and laser power on the dimensional accuracy of the obtained mesh structures were evaluated. The results showed that the heat of the molten pool was rapidly dissipated by the copper plate and the thin-layer structures were prevented from sticking to the surface (Figure 11b). In the experiments conducted with a powder layer thickness of 0.5 mm and a laser power of 200 W, a homogeneous fusion and a smooth layer formation were achieved. In addition, in the scanning path analysis of the mesh structures, it was determined that the straight scanning path reduced the residual stress and increased the material stability. It was observed that mesh structures of various sizes and shapes could be produced in a single step with this method. The study highlights the potential use in engineering applications such as filtration systems and liquid permeable components. Additionally, it was stated that surface quality could be improved by laser remelting process [168]. Luca Giorleo and Kudret İrem Deniz investigated the performance of molds produced with the 3D printing method for the steel bending process using polymer composite materials. This study compared horizontally and vertically-oriented molds produced using the Fused Filament Fabrication (FFF) method in order to reduce production costs and develop customizable molds for complex geometries. AISI 304 stainless steel sheets (100 mm length, 2 mm thickness, 20 mm width) and a “V” shaped punch and matrix produced from “Onyx” material with nylon-carbon fiber filling content were used. The production of the parts was done with two different printing orientations, horizontal (H) and vertical (V), with a 0.1 mm layer thickness. A total of 10 bending processes were performed for each orientation. Steel sheets were bent to a 90° angle with a hydraulic press at 1 mm/s speed (Figure 11c). The geometric measurements of the bent sheets and dies were analyzed using a coordinate measuring machine (CMM) over 180 data points. As a result of the study, the dies in the horizontal printing orientation showed deviations from the desired angle in some tests. For example, the angle was measured as 89.69° in the fifth test. The dies in the vertical printing orientation showed more stable performance with an angle deviation of 0.3%. In the horizontal dies, the punch height was reduced by 2.4% and the matrix width was widened by 10%. Horizontal printing lasted 1 day and 14 h, while vertical printing lasted 2 days and 4 h. This study showed that polymer-based dies are applicable for prototype production in steel bending processes. The vertical printing orientation provided higher stability, while the horizontal orientation shortened the production time [169]. Wang et al. produced materials using the selective laser melting (SLM) method by adding nickel (Ni) nanoparticles to AlSi10Mg alloy at different ratios (0%, 1%, 3%, 5%) and investigated their effects on microstructure and mechanical properties. The study analyzed the formation of in-situ Ala3Ni nanoparticles and Si network structures. The obtained structures consisted of α-Al matrix, Si network and Al3Ni phases. The increase in Ni content increased the continuity of Si networks, but microstructure deterioration was observed at 5% Ni level. The sample containing 3% Ni showed the best mechanical performance with 401 MPa tensile strength, 6.23% elongation and 144 HV microhardness value. This was attributed to the strength increase due to the strong interfacial bonding and dislocation accumulation of Al3Ni nanoparticles. At 5% Ni content, excessive energy absorption in the melt pool caused a “balling” effect and increased porosity, which reduced the strength. In the friction coefficient and wear tests, the samples containing 3% Ni exhibited the best performance with low wear volume (0.11 mm^3^) and stable friction coefficient (0.608). This study shows that Ni addition has the potential to improve the mechanical properties in AlSi10Mg alloy and excellent strength and wear resistance are achieved with an optimum 3% Ni content. These findings indicate important applications for the production of lightweight and durable structures, especially in the aerospace and automotive industries [170].

Katsuyama et al. investigated 3D carbon (3D-C) lattices fabricated using the low-cost stereolithography (SLA) type 3D printing method and carbonized via the pyrolysis process for structural lithium metal batteries. The research evaluated the mechanical strength of these carbon skeletons and their potential to enhance battery performance. The 3D-C lattice showed high mechanical stability with a maximum stress of 5.15 ± 0.15 MPa and Young’s modulus of 81.2 ± 6.4 MPa. SEM analysis showed that surface roughness due to AM was minimal and excellent contact was achieved between Li metal and the carbon skeleton. In battery performance tests, 3D-C lattices exhibited much more stable cycling stability compared to bare Cu foil collectors in symmetric cell configurations. When using the 3D-C cage, only 0.073 V low overpotential was observed for 3 mAh cm^−2^ capacity at 1 mA cm^−2^ current density for 100 cycles. In contrast, the overpotential in bare Cu foil cell reached 0.74 V at cycle 96 and failed due to a short circuit. The pores in the internal structure of the 3D-C skeleton prevented dendrite formation by controlling lithium accumulation and extended cell life. This study showed that 3D carbon skeletons have significant potential for increasing energy density, improving safety and providing lightweight structures in structural lithium metal batteries [173]. Ozlati et al. produced stainless steel (AISI 316L) and aluminum (AA5183) bi-metal walls using roll bonding transition joints using the WAAM method. The study investigated the effect of the transition joint, which prevents the formation of brittle Al-Fe intermetallic compounds by preventing direct contact between the steel and aluminum layers. Aluminum layers were deposited at low (LH), medium (MH) and high (HH) heat input levels (Figure 11d). The results showed that no intermetallic compounds were formed at the steel/aluminum interface and no cracks were observed when low heat input (LH) was used. The maximum tensile strength was measured as 42 MPa and the elongation was 21% in these samples. It was found that at medium and high heat input levels (MH and HH), the Al-Fe intermetallic compounds thickened and cracks formed at the interface. This was associated with increased thermal stresses and decreased material deformation. The HH sample, which was applied with high heat input, showed fracture during sample preparation during mechanical testing. The study revealed that low heat input limits the formation of Al-Fe intermetallic compounds in the WAAM process and improves mechanical performance. These findings highlight the potential use of the WAAM method in the production of bi-metal components with complex geometries such as heat exchangers in the energy sector [171]. Yang et al. developed an analytical temperature-based thermomechanical (ATTM) finite element (FE) model to predict residual stresses and deformations in the metal AM process. The model uses an analytical temperature approach to predict the temperature history and uses this thermal field as input data for mechanical calculations. The accuracy of the ATTM model was evaluated by comparing it with experimental data and other models in the literature. In the study, the simulations performed with the ATTM model accurately captured the temperature, stress, and deformation distributions for different geometry and material parameters of layered structures. The model was especially successful in studying the stress oscillation and thermal behavior of thin-walled and large-sized parts such as complex lattice structures. In addition, a simulation tool called AM-builder was developed in the study, which automatically generated process parameters by slicing the 3D model geometries according to the layer thickness. The ATTM model significantly reduced the computational time while providing high accuracy in mechanical calculations. In particular, the model’s prediction accuracy increased when the heat source was moved away from the boundary in the AM process. This approach offers a promising method to optimize temperature and mechanical stress analyses in AM processes [174]. Latte et al. developed a monitoring system using a prototype laser line scanner (LL) for the evaluation of layer and trace quality in the laser metal deposition (LMD) process. The study determined key performance indicators (KPIs) with a special data processing algorithm to detect problems such as geometrical defects and substrate porosity. Samples produced with AISI 316L stainless steel powders were deposited at different overlap ratios (10%, 30% and 50%). The prototype LL system scanned the layer surface using a laser line scanner and a CMOS camera to obtain the 3D profile of each layer. It was observed that the layer heights and contact angle values increased as the overlap ratio increased. Especially in the sample with a 50% overlap ratio, the contact angle reached a critical level of 55.8°, which led to porosity formation between the layers. However, the sample with a 10% overlap ratio showed mechanical risks due to deep valleys and surface waviness. Metallographic analysis results showed that the predictions obtained with the LL system were accurate and the proposed model was effective in detecting substrate defects. This study revealed that quality control can be performed during production without the need for destructive tests with laser-based monitoring systems [175]. Li et al. investigated the microstructure, mechanical strength and thermal expansion properties of structures produced using the laser metal deposition (LMD) method by adding TiC ceramic particles to Invar 36 alloy. In the study, TiC ratios ranging from 0% to 7% were used and the SEM, EBSD and XRD methods were used for microstructure analysis. The results showed that TiC addition significantly reduced the grains and increased the tensile strength. Between 0.8% and 2% TiC content, the tensile strength increased from approximately 130 MPa to 560 MPa, and the elongation ratio increased from 2% to 35%. However, at a TiC content above 3%, powder clusters were formed, which caused increased porosity and increased brittleness (Figure 11e). XRD analyses showed that the phase was too little to be detected at low TiC ratios, but the TiC phase became apparent after 3%. In thermal expansion tests, it was observed that the expansion coefficient of TiC-added samples increased, but the total expansion was lower than alloys commonly used in engineering applications. In particular, the expansion behavior was controlled in the region up to the Curie temperature. The study revealed that the addition of TiC to the Invar 36 alloy increased the mechanical strength and thermal stability, increasing its potential for use in aerospace and precision measurement devices [172]. Wu et al. aimed to increase the mechanical strength of bionic ceramic–metal composites using AM techniques. In this study, bionic structures inspired by tortoise shell structures in turtle shells and the deep-sea mollusk Crysomallon squamiferum were produced using the photopolymerization (VPP) and powder bed fusion (PBF) methods. The composites were reinforced with epoxy resin infiltration onto the Al_2_O_3_ ceramic base and a 316L stainless steel metal layer. Microstructure analyses were performed with SEM and EDS, and mechanical strength tests were performed with three-point bending tests. The results showed that the bionic layered structure increased fracture toughness by 41.83% and flexural strength by 41.79%. In particular, the 3D bionic structure increased the load-carrying capacity and prevented crack propagation thanks to the physical interlocking of the layered ceramic and metal phases. The finite element method (FEM) and molecular dynamics (MD) simulations detailed the load distribution and fracture mechanisms. It was found that bionic structures increased the energy absorption capacity by 554.94% compared to homogeneous structures. This study revealed that a 3D layered bionic structure has significant potential in the design of high-performance composite materials by increasing fracture strength and energy absorption [176].

### 4.3. Composite Materials

**Figure 12:**
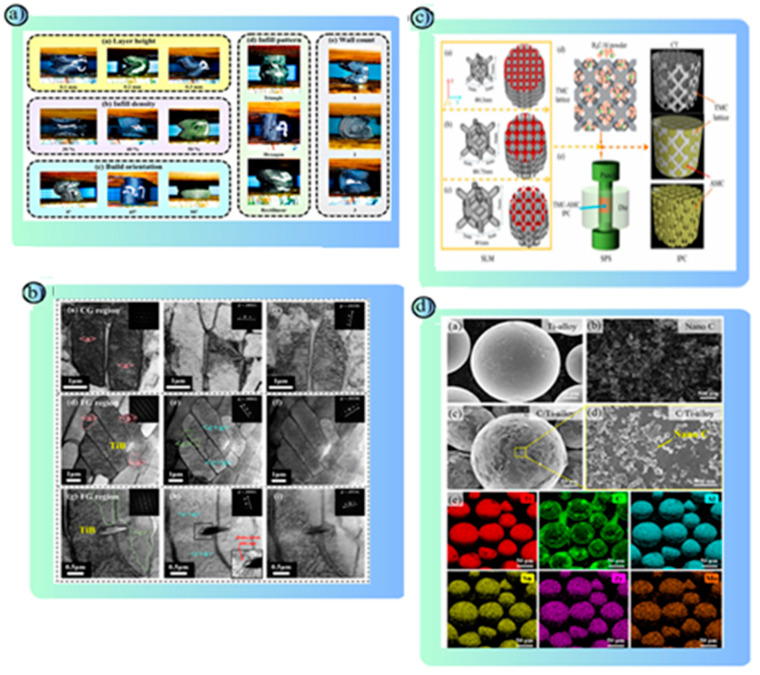
Composite figure showing various experimental analyses and methodologies related to advanced materials and AM: (**a**) Effects of layer height, infill density, structure orientation, infill patterns, and number of walls on the fractography modes of lattice-structured Onyx™ composites show changes in mechanical behavior [185]. (**b**) TEM images showing dislocation accumulation in coarse-grained (CG) and fine-grained (FG) regions under certain conditions, with detailed analysis of TiB regions and crystallographic orientations [189]. (**c**) Schematic representation of the preparation of TMC-AMC intercalated phase composite materials including 3D printing, filling of lattice pores with composite powders, and subsequent fabrication process via SPS, together with XRT images of the lattice and composite structures [190], and (**d**) morphological and elemental analysis of powders used in composite manufacturing, including alloy powders, nano carbon powders, C–Ti alloy composite powders and associated EDS maps showing elemental distributions [191].

Composite materials used in AM provide superior mechanical performance, lightness and durability by combining the advantageous properties of different materials [177,178]. Composite materials are generally composed of a matrix (polymer, metal or ceramic) and a reinforcement element (fiber, particle or sheet) to optimize critical performance parameters such as flexibility, energy absorption capacity and impact resistance. Polymer matrix composites (PMC) stand out with their high strength-to-weight ratio, corrosion resistance and ease of production [179]. Carbon fiber and glass fiber reinforced polymers are widely used in barriers and crash buffer systems, increasing performance and reducing material waste by enabling production in complex geometries through AM [180]. Metal matrix composites (MMC), on the other hand, offer high-temperature resistance and strength, and offer high resistance to impacts and long life, especially thanks to the reinforcement of aluminum, titanium and magnesium matrices with ceramic or carbon fiber reinforcements [181]. Ceramic matrix composites (CMC) have been developed for applications requiring high temperature and wear resistance, and by strengthening with fiber or particle reinforcements, mechanical brittleness is reduced and energy absorption capacity is increased [182]. It is especially preferred in critical structural components such as fasteners or barrier segments exposed to heat. Hybrid composites, on the other hand, offer both cost advantages and high impact resistance as special systems where more than one matrix or reinforcement material is used together allow for the combination of different functional areas in a single structure and design flexibility with AM. Additive manufacturing technologies increase the bond strength between layers by ensuring that composite materials are used in accordance with the layered manufacturing principle and offer the opportunity to produce complex geometric structures at low cost. Structures designed with composite materials provide significant advantages in road safety systems thanks to their lightness and high energy absorption capacity. There are extensive studies in the literature on the integration of composite materials with AM. These studies provide important data to improve the performance of road safety structures, and research in this field progresses dynamically and continuously develops. Garmabi et al. conducted studies on the 3D printing of polyphenylene sulfide (PPS) composites reinforced with recycled carbon fiber (rCF) for use in the automotive industry. The study evaluated the mechanical, thermal and chemical resistance properties of composites containing 5% and 10% rCF. According to the results, a 10% rCF addition increased the elastic modulus by 78%, impact strength by 64% and collapse strength by more than 60% compared to pure PPS. In addition, the degradation temperature of the components increased by approximately 50 °C. When the manufactured parts were exposed to automotive fluids (gasoline, diesel and coolant) for a week, the weight change remained below 1% and the tensile strength decreased by only 10%. SEM analyses showed that rCFs were homogeneously distributed in the matrix, but fiber pullout occurred in some samples due to fiber-matrix incompatibility. In rheological tests, a viscosity increase was observed at a low frequency, while a decrease was observed at a high frequency; this shows that printing ability is improved. Thermal analyses revealed that rCF reinforcement increases the overall thermal stability of the composite despite reducing crystalline regions. In flame retardancy tests, UL94-V0 level of fire retardancy was achieved. Finally, an automobile radiator coolant pipe prototype was produced with the developed filaments and it was observed that the obtained parts have industrial quality. This study shows that PPS composites produced with recycled carbon fiber are potential candidates in the production of sustainable and lightweight structures in the automotive sector with their high-temperature resistance and mechanical strength [183]. Zhai et al. investigated the effect of 3D printing process parameters on mechanical properties of fiber-reinforced thermoplastic composites. The study evaluated how continuous fiber-reinforced composites produced using the Fused Filament Fabrication (FFF) method can be optimized with parameters such as printing temperature, printing speed, layer thickness and printing path. It was observed that fiber-reinforced composites exhibited superior mechanical properties compared to short fiber reinforcements, but there were difficulties such as fiber placement and void formation in the printing of complex shapes. High printing temperatures increased interlayer adhesion, while excessive temperatures caused deformations. Increasing printing speed decreased adhesion quality, but appropriate speed parameters provided balance in printing time and product quality. Thin layer thickness provided better tensile strength, while thick layers increased flexural strength. Fiber orientation and printing path were found to have a direct effect on the load-carrying capacity and durability of the products. The study highlights the importance of optimizing process parameters to improve the mechanical performance of 3D-printed products and suggests that AI-based control systems could revolutionize this field in the future [184]. Sivakumar et al. investigated the effect of material extrusion (MEX) parameters on the compression properties of honeycomb lattice structures of carbon fiber-reinforced Onyx™ composites. In the study, the effect of parameters such as layer height, filling ratio, structure orientation, infill pattern and number of walls on physical and mechanical properties such as compression strength, density and diameter deviation were evaluated (Figure 12a). The highest compression strength in triangular lattice structures of Onyx™ composites produced using the Fused Filament Fabrication (FFF) method was obtained with 0.1 mm layer height, 50% filling ratio, 90° structure orientation, rectangular infill pattern and three walls. While the increase in layer height caused a decrease in compression strength, lower layer height provided stronger interlayer adhesion. In addition, with a 90° structure orientation, load-carrying capacity increased since the material layers were placed vertically, whereas lower strength was recorded with 0° and 45° orientations due to layer slippage. Rectangular patterned infill provided high strength by increasing the filling density, while triangular and hexagonal patterns created voids, which reduced strength. In three-walled samples, it was observed that deformation occurred more slowly, and energy absorption increased. The study highlighted the potential of Onyx™ composites for use in engineering applications such as lightweight and high-strength camera holders, and showed that these structures provided 23% weight savings and 15% mechanical improvement [185]. Yu et al. investigated tungsten carbide (WC)-reinforced iron matrix composites manufactured using the WAAM process. The study investigated the manufacturing mechanism of the composites using an innovative system in which WC particles were fed with powder using gravity and the effects of WC particle size on the microstructure, mechanical properties and wear resistance. Thin-walled samples were produced using WC powders of different sizes (106 µm and 250 µm). WC was added at 40 wt% in the AM with low carbon steel (Q235B) base material and ER70S-6 wire. A microstructure analysis showed that small WC particles were concentrated in the center, while large particles were more homogeneously distributed. Large WC particles were found to be more resistant to melting in the melt pool and resulted in the formation of wider but thin-layered weld beads. According to the tensile test results, in samples with small WC particles, yield strength increased by 80%, ultimate tensile strength increased by 70%, while ductility decreased (from 41.9% to 6.9%). In large particle samples, tensile strength is lower due to microcrack formation. Microhardness tests showed that WC reinforcement increased hardness by 130%. As a result of wear tests, wear loss in samples containing WC decreased by 40%. It was determined that the wear mechanism occurred with primary adhesive wear and light abrasive wear. This study made a significant contribution to understanding the distribution behavior of WC particles in the production of iron matrix composites with the WAAM method and offered the potential for low-cost production of large components [186]. Landrie et al. examined the ballistic resistance properties of thermoplastic composite plates produced using extrusion AM. In the study, carbon fiber-reinforced polycarbonate (PC-CF), glass fiber-reinforced polycarbonate (PC-GF), thermoplastic polyurethane-acrylonitrile butadiene styrene/carbon fiber (TPU-ABS/CF) and acrylonitrile butadiene styrene-carbon fiber (ABS-CF) composites were tested at different parameters). In particular, the effect of nickel-chromium wire-reinforced PC plates on energy absorption and hole opening mechanisms was evaluated. The tests were carried out with a gas gun reaching a bullet speed of up to 400 m/s. According to the results, nickel-chromium wire-reinforced samples absorbed 14–17% more energy than unreinforced samples. TPU-ABS/CF samples exhibited a mechanism to prevent bullet passage by changing the deformation path and thanks to the flexibility of the material, thus, minimum damage was observed at the exit point. ABS-CF composites showed the highest strength by absorbing 94.5% of energy. Detailed examinations of damage mechanisms such as delamination and fiber pullout were made with scanning electron microscopy (SEM). The study showed that large-scale composite panels have high ballistic protection potential and offer an innovative alternative that can be used especially in military and infrastructure applications [187]. Chaves Figueiredo et al. developed the mechanical behavior of stress-hardening cementitious composites (SHCC) printed using the layered manufacturing method. The study evaluated the anisotropic behaviors in the mechanical performance of fiber-reinforced cementitious composites in relation to the printing direction. The SHCC mixtures used were reinforced with polyvinyl alcohol (PVA) fibers at a 2% volume ratio and hydroxypropyl methylcellulose (HPMC) additive was added to control the fluidity. The samples were printed with different layer heights and subjected to mechanical tests. In the compressive strength tests, the performance was compared by applying loading parallel and perpendicular to the printing direction. The results showed that a strong bond was formed between the layers and the composite exhibited high strength even under loading perpendicular to the printing direction. In the flexural strength tests, samples with different numbers of layers were examined and lower ductility was observed in thicker samples. Uniaxial tensile tests revealed that aligning the fibers parallel to the stress direction increased the load-carrying capacity of the material, but reduced the strength in other directions. Micro CT scans confirmed that the fibers were largely aligned parallel to the stress direction and showed that air gaps affected the orientation of the fibers, reducing mechanical performance. This study highlighted that 3D-printed SHCC materials can offer high mechanical strength and ductility with appropriate stress parameters and material design, and suggested potential applications in the construction industry [188].

Sikora et al. conducted a review study examining the effects of nano- and micro-sized additives in cementitious and alkali-activated composites suitable for 3D printing. The study evaluated the effects of nano and micro particles on fresh and hardened material properties, especially in concrete mixtures. The effects of additives such as nanosilica, graphene-based materials and clay nanoparticles on rheological behavior and mechanical strength were discussed in detail. The accelerating effects of nano additives on the cement hydration process and the potential to improve thixotropic properties are important in terms of strengthening the interlayer bond during printing. As a result of the study, it was emphasized that nano additives significantly improve the fluidity and adhesion properties of fresh mixtures even at low doses, but may lead to extrusion difficulties and weak interlayer bond formation at high dosages. It was determined that nanosilica and nanoclay additives in particular strengthened the thixotropic structure and provided a significant increase in the early age strength of nanosilica. In addition, graphene-based materials have increased strength through the crack-bridging mechanism by offering high elastic modulus and tensile strength. The study suggests that optimal dosages of nano-additives should be determined and more comprehensive research is needed on the durability performance of 3D printed concretes in the future [192]. Feng et al. investigated the double heterogeneous titanium matrix composites produced by layered manufacturing using self-assembled powders enriched with nano-reinforcements. In the study, a structure containing micro-scale networks and grain size gradients formed by Ti6Al4V alloys and TiB and TiC nano-reinforcements was designed. Laser-directed energy deposition (LDED) method was used in the layered manufacturing process and the reinforcements were kept in nano-size thanks to the rapid solidification. In the microstructure analyses, it was observed that network structures were formed around the previous β grains in the composites and TiB-TiC reinforcements offered a unique load transfer capability. In mechanical tests, samples with 1.5% reinforcement ratio reached 1071 MPa tensile strength with 13.25% elongation and had 32% higher ductility compared to the conventional Ti6Al4V alloy. Increasing reinforcement ratios showed a decrease in ductility due to deformation mismatches leading to microcrack formation. Electron microscope analyses showed that dislocations accumulated along the network structures and heterogeneous deformation-induced hardening occurred in these regions (Figure 12b). The study revealed that double heterogeneous titanium composites offer a new approach for improving the balance of strength and ductility and may be a promising solution for aerospace, automotive and biomedical applications [189]. Xiong et al. fabricated a dual-phase composite by infiltrating B4C/AlSi10Mg into the CoCrFeNiMn/Ti6Al4V lattice structure using laser 3D printing and spark plasma sintering (SPS) techniques (Figure 12c). The study investigated the effects of biomimetic dual-phase structures on impact strength and energy absorption. The results showed that the complex dual-phase structure and the interfacial bonding mechanism effectively ensured load transfer by preventing local damage propagation. The cracks in the CoCrFeNiMn/Ti6Al4V phase were mechanically locked by infiltrating B4C/AlSi10Mg, and the damage tolerance of the composite was increased. Compression tests showed that cell size and lattice diameter played an important role in the structure’s durability. In particular, composites with 4 mm cell size (IPC-4) exhibited high energy absorption performance with 566 MPa maximum compression strength and large deformation capacity. Dynamic loading tests revealed that thermal softening and hardening behaviors are in competition with increasing temperature and strain rate. Ti3AlSi5 phase formed at the interface contributed to the strengthening of the metallurgical bond as well as the mechanical bond and increased the load transfer. This structure is promising for impact-resistant lightweight structure applications in sectors such as the defense industry and automotive with its superior strength, ductility and energy absorption capabilities [190]. Mingione et al. produced polyamide 12 (PA-12) composites reinforced with graphene nanoplates (GnPs) using the selective laser sintering (SLS) method and evaluated the thermal, electrical and tribological performances of these materials. In the study, composite samples containing GnPs additives at different weight percentages (2%, 4%, 6%, 8% and 10%) were produced and their performances were compared to the pure PA-12 matrix. As a result of the tests, the electrical conductivity increased from 10⁻^11^ S/cm to 10⁻^4^ S/cm with 10% GnPs additive, providing an eight-step increase in conductivity. Thermal performance tests showed a 33.6% improvement in the time to reach 90 °C. In tribological tests, the friction coefficient decreased by 25%, while the wear volume decreased by 81%. Surface analyses revealed that the GnPs additive did not negatively affect the surface quality, but increased porosity was observed with increasing additive rates. In hydrophobicity tests, the contact angle increased by 43% and the surface gained a hydrophobic character. This study showed that GnPs reinforced PA-12 composites have functional properties that can be used in low-cost production processes and are promising for applications such as electronic device housings, low-friction gears and bearings [193]. Cuccarollo et al. carried out the characterization and modeling of the microstructural properties and mechanical behaviors obtained as a result of the production of continuous fiber-reinforced polymer composites (C4F) using the AM method. In the study, carbon fiber/polyamide 12 (CF/PA12) composite samples were tested in order to investigate the effects of production parameters and fiber trajectories on different load orientations. The orientation of continuous fibers according to the load path was optimized by reducing the stress concentrations in the holes and recesses where the load concentrations occur. In the produced samples, it was observed that interlayer porosity and fiber alignment defects negatively affected the mechanical strength. In the microstructure analyses, it was determined that the fiber density was 50% and the void ratio in the structure was kept below 5%. After the thermal treatment, the tensile strength reached 1091 MPa with the strengthening of the interlayer bond. The elastic properties were estimated using finite element modeling and the Mori–Tanaka homogenization approach and results compatible with the experimental data were obtained. This model provides flexibility in the analysis of components with different fiber arrangements and geometric properties, providing an innovative approach that can be used in design strategies for lightweight and durable structures [194]. Wang et al. investigated the effects of process parameters on microstructure and mechanical properties of TiC/Ti alloy matrix composites produced using the laser directed energy deposition (DED) method. In the study, TiC-reinforced composite powders were produced using Ti-5.6Al-4.8Sn-2.0Zr-1.0Mo-0.85Nb-0.34Si alloy and nano-sized carbon powder (Figure 12d). Microstructure and phase analyses of samples produced using the DED method at different currents and scanning speeds were performed using X-ray diffraction (XRD), scanning and transmission electron microscopy (SEM/TEM). It was determined that the sizes of TiC particles increased with increasing current values, but they were distributed evenly in the matrix at low scanning speeds. In tensile tests, the tensile strength of composite samples produced at a 40 A current and 3 mm/s scanning speed was found to be 1412 MPa, 28% higher than the wrought Ti alloy. High energy density caused the TiC nucleation sites to decrease but grow, and the phase morphology changed from thin lamellar to blocky α phase. Microstructure analyses showed that TiC particles provided increased strength by preventing dislocation movement within the matrix. This study demonstrates that TiC/Ti alloy composites produced using the DED method provide high mechanical strength while maintaining ductility and have significant potential for aerospace, automotive, defense and biomedical applications [191]. Li and et al. examined the biomimetic folding mechanisms of polycarbonate (PC)-based ternary composites produced using 4D printing. In the study, a composite that responds to thermal and electrical stimuli was designed by reinforcing polycarbonate, carbon nanotubes (CNTs) and carbon fiber (CF), which offers high-temperature resistance and excellent shape memory properties. This structure increased electrical conductivity by creating a “dot + line” conductive path and allowed the material to reach 150 °C at its extreme points within 1 min under 50 V voltage without affecting the shape memory performance. The shape recovery rate reached 100% in U-shaped samples and no structural deterioration was observed for 20 cycles. Folding and opening mechanisms in nature such as mimosa, sunflower and butterfly wings were simulated in biomimetic model designs. The mimosa model returned to its original shape in 2 min and the butterfly model in 3 min. Experiments have shown that the rigidity and load-carrying capacity of U-shaped samples are optimized to provide energy absorption. This study provides an important step for stimulus-responsive foldable structures and secondary opening mechanisms in space applications such as solar panels [195]. Zhang et al. investigated the fabrication of pyramidal honeycomb PEEK/CF composite metastructures optimized for electromagnetic wave (EMW) absorption. In the study, a design that provides high-performance EMW absorption in a lightweight and thin structure was realized by combining the advantages of honeycomb and pyramid geometries. The metastructure produced using the FDM method using PEEK/CF filament was coated with carbonyl iron powder (CIP) sputtering process. The EMW absorption mechanism was supported by electrical and magnetic losses as well as the interfacial polarization effect. The results showed that the pyramidal honeycomb structure provided a wide effective absorption bandwidth (EAB) in Ku, K and Ka bands (12.44–40 GHz) and exhibited high performance at angles up to 45°. Optimization of design parameters was performed with factors such as honeycomb cell diameter (Lh), structure height (H) and periodic length (Lp) and ideal structure parameters were determined as Lp = 20 mm, Lh = 5 mm and H = 10 mm. Electric field simulations revealed that there is a strong magnetic field distribution in pyramidal regions and that cyclic current loops (SCLC) contribute to EMW absorption in these regions. Experiments showed that the optimized structures are insensitive to azimuthal angle and provide effective absorption in TE-TM modes. This study emphasizes that lightweight metastructures that can be produced with 3D printing have wide application potential in the aerospace and defense sectors [196].

## 5. Challenges and Future Perspectives

AM technologies are gaining attention for their design flexibility, customizability, material efficiency and sustainable production possibilities in road safety systems. However, there are also some significant challenges that limit the widespread and industrial-scale applicability of these technologies [197,198]. Especially in high-volume manufacturing processes, the unit production costs of AM can be higher compared to conventional methods. This is due to the high cost of raw materials such as metal powders or specialty filaments, the slow pace of the printing process and additional post-processing requirements. However, for low-volume or complex geometry production, AM can offer a more economical and efficient alternative to conventional methods, thanks to the elimination of the need for molds and the minimization of material waste [199]. On the other hand, the range of materials that can be produced with AM is still limited and the machinability of some metal or multiphase composite materials, especially those used in high-performance engineering applications, is restricted. This necessitates caution in material selection for applications where structural strength is critical, such as road safety. Furthermore, the lack of internationally accepted testing protocols and certification processes to evaluate the performance of AM-produced structures is another important factor limiting the use of these technologies in infrastructure projects.

Furthermore, the absence of comprehensive regulatory frameworks and universally recognized standardization processes presents a significant impediment to the widespread industrial application of AM technologies, particularly in critical infrastructure sectors such as road safety systems. Given the safety-critical nature of infrastructure applications, including barriers, crash cushions, and protective elements, the lack of internationally agreed-upon testing protocols, performance standards, and certification procedures undermines the confidence required for large-scale adoption. The absence of regulatory consistency across regions also complicates the integration of AM components into public safety standards. Therefore, it is imperative for future research and collaborative policy efforts to focus on establishing unified, internationally accepted guidelines for testing, certification, and quality assurance in AM technologies. Such efforts will not only facilitate the seamless integration of AM in infrastructure but also enhance the reliability, safety, and cost-effectiveness of AM-produced components, thereby fostering wider acceptance and regulatory compliance in transportation and civil engineering domains. Therefore, future research should not only focus on material development and design optimization, but also on increasing production speeds, ensuring cost-effectiveness, enabling production with a wider range of materials, and improving standardization processes. This holistic approach will pave the way for the wider application of AM in road safety systems.

In the future, AM technologies are expected to find wider applications in transportation infrastructure and road safety systems. Several key innovation and research foci stand out in this process:

Smart and Sensor Integrated Systems: With sensor and IoT technologies integrated into the materials, smart road safety systems can be developed for real-time data collection and analysis. For example, it may be possible to instantly measure barrier deformation and the load on it during a collision and transmit it to traffic management systems.

Innovative Materials and Compounds: More widespread use of innovative materials such as biodegradable polymers, nanocomposites and hybrid materials in AM will improve environmental sustainability and enable the design of higher-performance road safety systems. Furthermore, high-tech materials, such as graphene-reinforced composites with advanced durability and energy absorption capacity, can be integrated into road safety systems.

Global Sustainability and Circular Economy: The reuse of waste polymers and recycled materials will reduce the carbon footprint of road safety systems and contribute significantly to environmental sustainability. Circular economy principles can be applied to the entire life cycle of road safety materials, from production to recycling.

Improvement in Manufacturing Technologies: By developing faster, cost-effective and more precise manufacturing processes, the applicability of AM systems on industrial scales can be increased. Energy-saving production methods and artificial intelligence-supported design algorithms will drive developments in this area.

Multidisciplinary Approaches: The integration of disciplines such as materials science, engineering, simulation technologies and data analytics can enable more efficient, durable and optimized AMed security systems.

In conclusion, AM technologies will continue to provide safe, durable, environmentally friendly and economical solutions for transportation infrastructure and road safety. Future research will contribute to making this technology more accessible and versatile, creating safer and sustainable infrastructures in urban and intercity transportation systems. These advances will create new opportunities not only for the transportation sector, but also for general industrial applications.

## 6. Conclusions

Each of the studies reviewed in this paper investigated the mechanical strength, energy absorption capacity and thermal properties of polymer, metal and composite materials, and optimized performances were achieved with different lattice structures, print orientations and reinforcement materials. Although not directly applied to the production of road safety structures, the results show that these materials can be used in critical structural elements such as road barriers, crash cushions and safety parapets. For example, by producing polymer-based barriers with the FDM method, energy absorption zones with different densities can be designed and reinforcement can be provided with thicker layers at critical points of the barrier. Using the SLA method, crash cushions with high-resolution and flexible geometries can be produced and deformation can be managed in a controlled manner during impact. The production of safety parapets from polymer composite materials with increased thermal resistance using the SLS method can offer long-lasting structures by increasing resistance to outdoor conditions. Analyses have shown that polymer composites are an ideal solution to increase energy absorption and mechanical strength. For example, it is understood that glass fiber or carbon fiber-reinforced polymer barriers can offer compressive strength of over 27 MPa and minimize deformation during impact. This type of structure can be 30–40% lighter than conventional steel or concrete barriers and can offer the advantage of easier portability and installation. Furthermore, polymer barriers manufactured using versatile lattice structures can significantly reduce the severity of second impacts by evenly distributing energy during a collision. Different types of materials such as polymers, metals and composites have been optimized to meet the critical requirements for energy absorption capacity, mechanical strength and longevity in road safety systems. For example, barriers made of polypropylene with recycled tire rubber achieved a 69% CO_2_ reduction and 86% less energy consumption. Barriers made with EVA foam cylinders improved crash performance by reducing impact energy by 30%. The three-wave steel barrier design absorbed 60% of the momentum, reducing the accident severity transferred to passengers. Composite corrugated movable steel barriers can provide fast safety measures on highways with an installation speed of 12 m/min and a closing speed of 2 min. Composite barriers made of recycled concrete and thin-walled steel reduced impact force by 54.46% and cost by 5.5%. The innovations enabled by AM have been particularly prominent in the design of barrier systems that can absorb impact energy and distribute deformation in a controlled manner. In conclusion, the findings of this study show that AM technologies have a strong potential to provide modular, durable and customizable solutions for road safety structures. These methods will not only improve the mechanical strength of safety structures, but also offer cost savings, sustainability and rapid prototyping advantages in the manufacturing process. It also reveals that AM offers benefits not only in terms of technical, but also economic and environmental sustainability. The use of recyclable materials and the reduction of waste in the manufacturing process minimize environmental impacts, while innovative designs that save energy have made this technology more attractive. AM appears to play a key role in the production of structures with complex geometries that are difficult or impossible to produce with conventional methods.

## Figures and Tables

**Figure 6:**
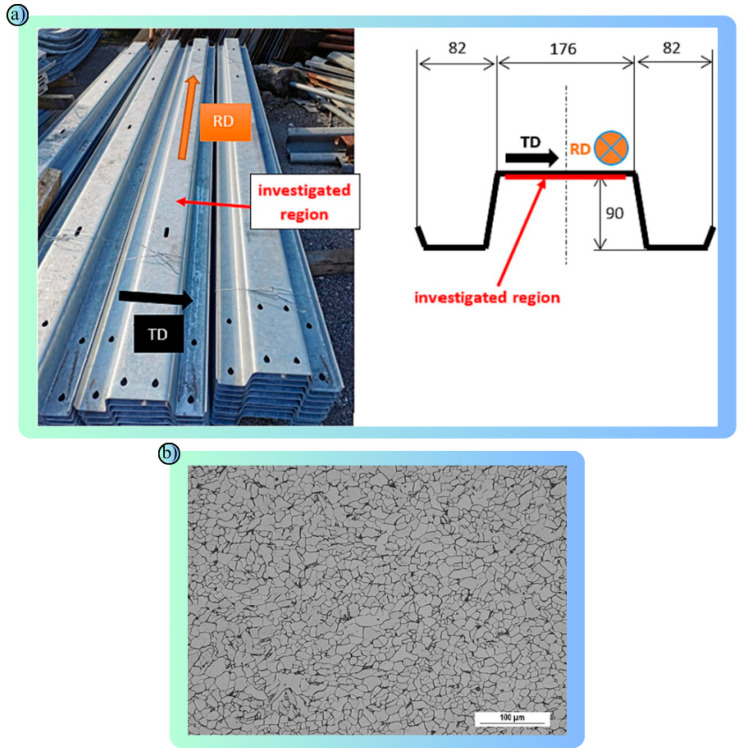
Comprehensive illustrative representation that brings together experimental data and modeling results from studies: (**a**) The analyzed region of the traffic barrier flanges and the orientation axes are shown: RD (Rolling Direction) and TD (Transverse Direction) are indicated and the region where the mechanical tests were performed is marked, (**b**) The sample shows a ferritic matrix structure and irregularly distributed pearlite islands: The 1% Nital etched microstructure was studied to evaluate the homogeneity and mechanical properties of the steel [92].

**Table 1:** Comparative overview of AM techniques.

Technique	Accuracy	Surface Finish	Material Range	Cost	Production Speed	Advantages	Limitations
FDM	Low–Medium	Rough	Thermoplastics	Low	Fast	Low cost, easy to use, rapid prototyping	Poor surface finish, limited material strength
SLA	High	Excellent	Photopolymers	Medium	Slow	High detail, smooth surfaces	Brittle parts, limited material types
DLP	High	Excellent	Photopolymers	Medium	Fast	High resolution, faster than SLA	Limited build volume, costly resins
SLS	Medium	Good	Polymers, Composites	Medium–High	Medium	No support needed, good mechanical properties	Rough surface, powder handling required
SLM	High	Good	Metals	High	Slow	High strength, dense metal parts	Expensive, requires post-processing
DMLS	High	Good	Metals	High	Slow	Excellent detail and strength	High cost, slow process
